# Supplementary material for: AAV capsids target muscle-resident cells with different efficiencies—A comparative study between AAV8, AAVMYO, and AAVMYO2
Source: Mol Ther Methods Clin Dev. 2025 Mar 14;33(2):101451. doi: 10.1016/j.omtm.2025.101451 (PMC11987650; doi:10.1016/j.omtm.2025.101451)
Supplement: Document S1. Figures S1–S7 and Tables S1–S8 [file mmc1.pdf]

OMTM, Volume *33*

Supplemental information

AAV capsids target muscle-resident cells with different efficiencies—A comparative study between AAV8, AAVMYO, and AAVMYO2

Timothy J. McGowan, Nicolas Lewerenz, Eleonora Maino, Marco Thürkauf, Lena Jörin, and Markus A. Rüegg

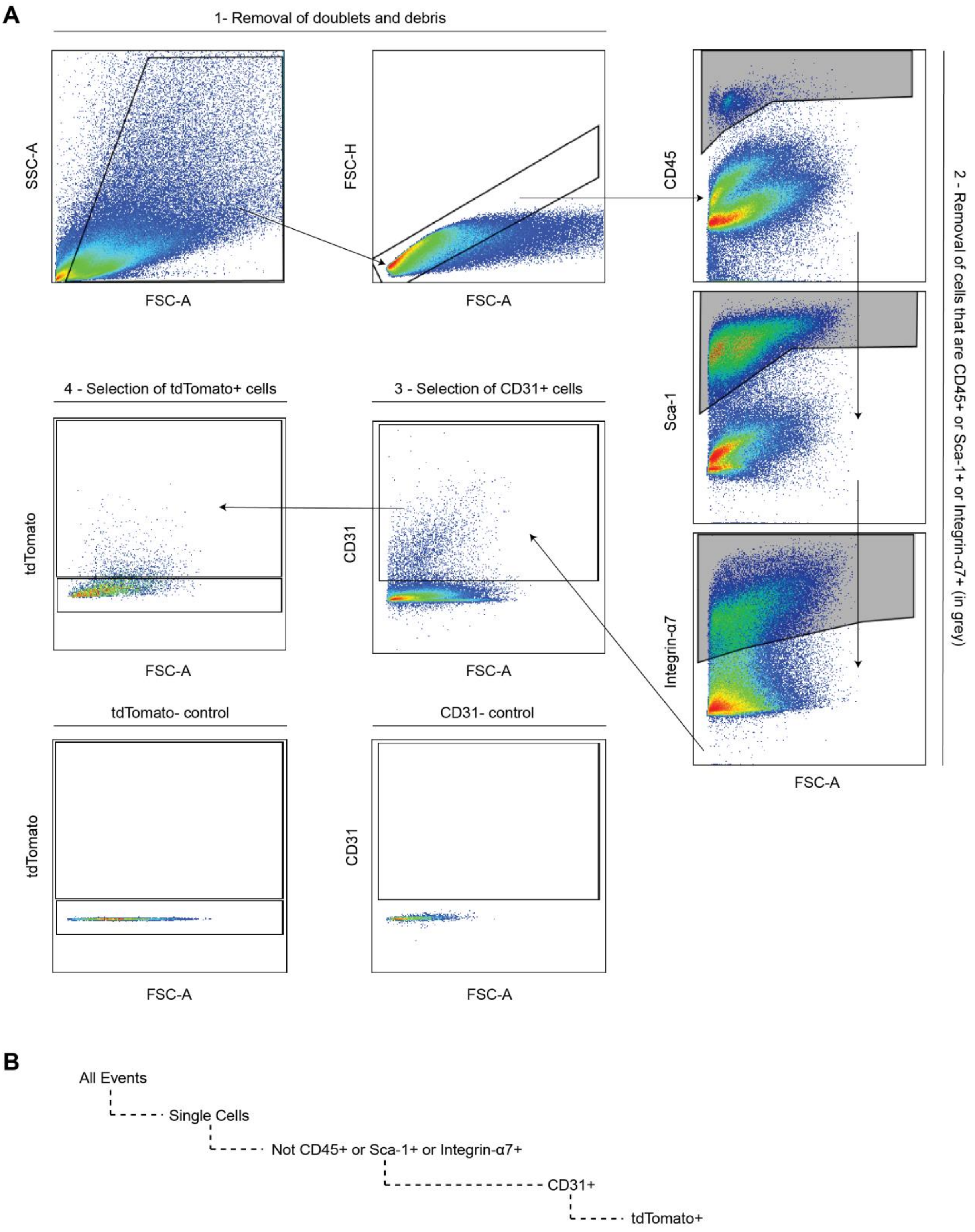
A
1- Removal of doublets and debris
SSC-A
FSC-A
FSC-H
FSC-A
CD45
Sca-1
Integrin-α7
FSC-A
2 - Removal of cells that are CD45+ or Sca-1+ or Integrin-α7+ (in grey)
4 - Selection of tdTomato+ cells
tdTomato
FSC-A
3 - Selection of CD31+ cells
CD31
FSC-A
tdTomato- control
tdTomato
FSC-A
CD31- control
CD31
FSC-A
B
All Events
Single Cells
Not CD45+ or Sca-1+ or Integrin-α7+
CD31+
tdTomato+

<u>Fig. S1: Design of the flow cytometry panel.</u> For visualization purposes, we show the flow cytometry panel design for one cell population (CD31+ cells). The following steps were applied for all cell populations. **A** We first removed cell duplicates and focused on single cells (1). Then, we removed cells that stained positive for CD45, Sca-1 or Integrin-α7 (2) and kept cells that were positive for CD31 (3). Finally, within this population, we quantified the proportion of cells that were tdTomato+ (4). A fluorescence-minus-one control in which CD31 antibody was omitted and a tdTomato-negative sample were used to set the boundaries between cells that were positive or negative for a marker. These controls are displayed under the panels showing the selection of CD31+ cells (3) and tdTomato+ cells (4), respectively. **B** Text description of the sorting strategy.

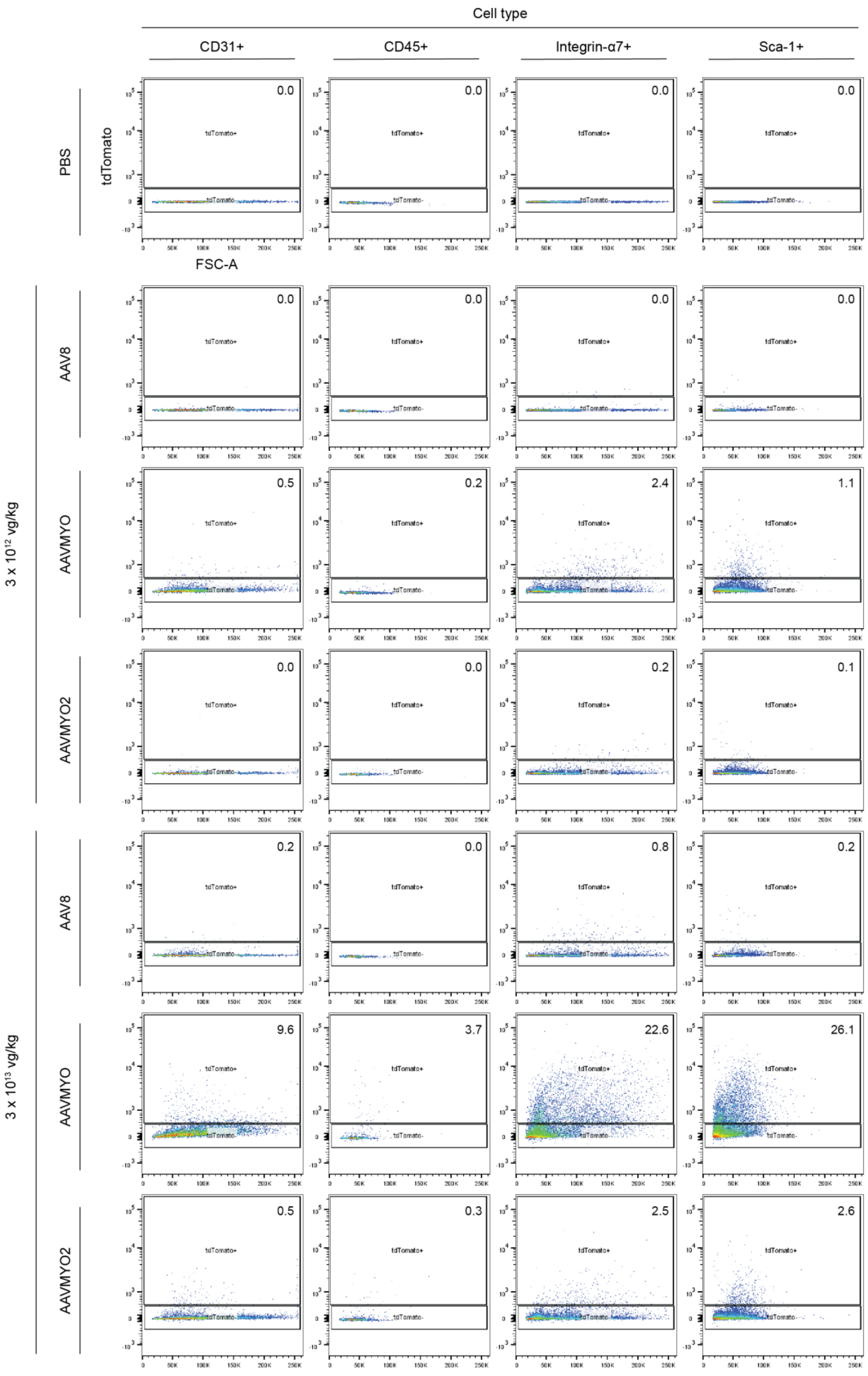
Cell type
CD31+
CD45+
Integrin-α7+
Sca-1+
PBS
AAV8
AAVMYO
AAVMYO2
3 x 10^12 vg/kg
3 x 10^13 vg/kg
tdTomato
FSC-A
tdTomato+
tdTomato-
0.0
0.0
0.0
0.0
0.0
0.0
0.0
0.0
0.5
0.2
2.4
1.1
0.0
0.0
0.2
0.1
0.2
0.0
0.8
0.2
9.6
3.7
22.6
26.1
0.5
0.3
2.5
2.6

Fig. S2: Flow cytometry panel after IV injection of AAV8, AAVMYO and AAVMYO2. Representative panels showing tdTomato signal in CD31+, CD45+, Integrin-α7+ and Sca-1+ cells (the staining strategy to isolate these cells is shown in Fig. S1) of mice injected IV with PBS, AAV8, AAVMYO or AAVMYO2 with 3.0 x 10^{12} or 3.0 x 10^{13} vg/kg. The average proportion of tdTomato+ cells is displayed in the top right corner of each panel.

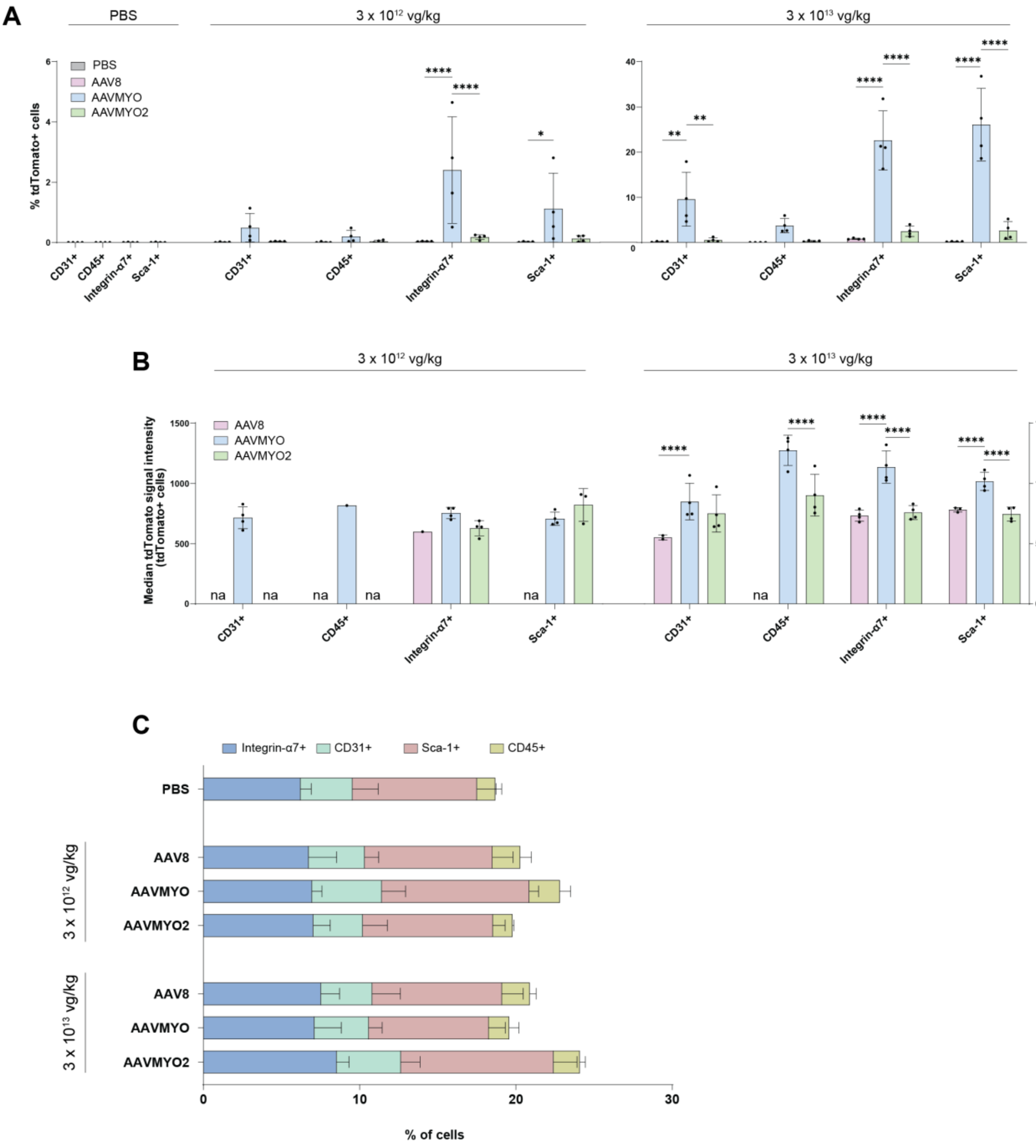
A
PBS
3 x 10^12 vg/kg
3 x 10^13 vg/kg
PBS
AAV8
AAVMYO
AAVMYO2
% tdTomato+ cells
CD31+
CD45+
Integrin-α7+
Sca-1+
B
Median tdTomato signal intensity (tdTomato+ cells)
na
C
Integrin-α7+
CD31+
Sca-1+
CD45+
PBS
AAV8
AAVMYO
AAVMYO2
% of cells

Fig. S3: Transduction rates and effect on muscle composition of IV-injected AAV8, AAVMYO and AAVMYO2. **A** Quantification of the proportion of tdTomato+ cells in CD31+, CD45+, Integrin-α7+ and Sca-1+ cells. **B** Quantification of the median tdTomato signal intensity in CD31+/tdTomato+, CD45+/tdTomato+, Integrin-α7+/tdTomato+ and Sca-1+/tdTomato+ cells. na = not applicable, when < 5 tdTomato+ cells were detected in the cell population, the median tdTomato signal was not measured. **C** Quantification of the proportion of CD31+, CD45+, Integrin-α7+ and Sca-1+ cells detected during flow cytometry. n = 4. Data are shown as means ± SD. In **A** and **B**, statistics were evaluated using one-way ANOVAs with Tukey's multiple comparisons test. In **C**, statistics were evaluated using two-way ANOVAs with Tukey's multiple comparisons test. $p < 0.05$ = *, $p < 0.01$ = **, $p < 0.001$ = ***, $p < 0.001$ = ****.

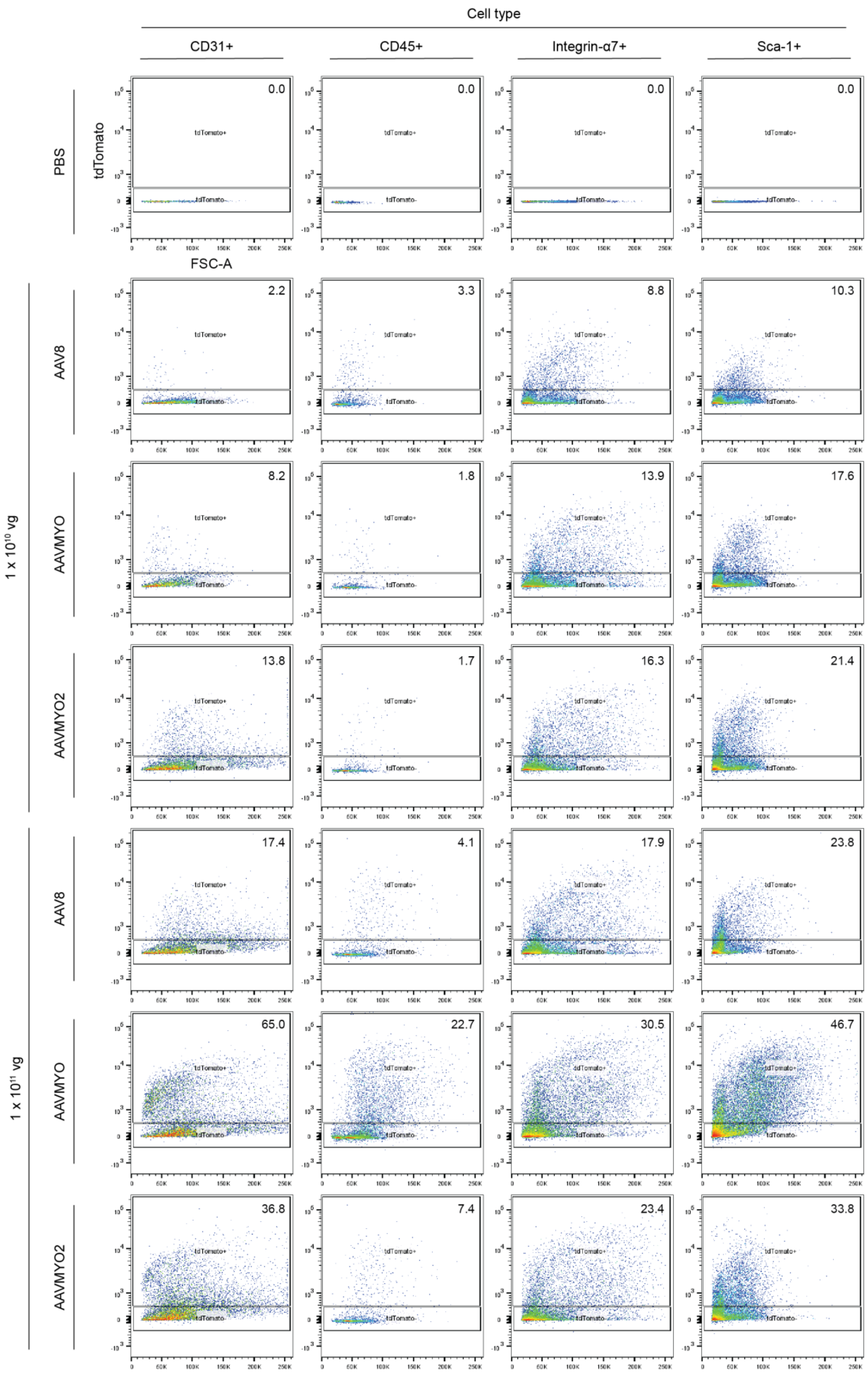

Cell type
CD31+
CD45+
Integrin-α7+
Sca-1+
PBS
tdTomato
FSC-A
1 x 10¹⁰ vg
1 x 10¹¹ vg
AAV8
AAVMYO
AAVMYO2
tdTomato+
tdTomato-
0.0
0.0
0.0
0.0
2.2
3.3
8.8
10.3
8.2
1.8
13.9
17.6
13.8
1.7
16.3
21.4
17.4
4.1
17.9
23.8
65.0
22.7
30.5
46.7
36.8
7.4
23.4
33.8

<u>Fig. S4: Flow cytometry panel after IM injection of AAV8, AAVMYO and AAVMYO2.</u> Representative panels showing tdTomato signal in CD31+, CD45+, Integrin-α7+ and Sca-1+ cells (the staining strategy to isolate these cells is shown in Fig. S1) of mice injected IM with PBS, AAV8, AAVMYO or AAVMYO2 with 1.0 x 10^{10} or 1.0 x 10^{11} vg per TA. The average proportion of tdTomato+ cells is displayed in the top right corner of each panel.

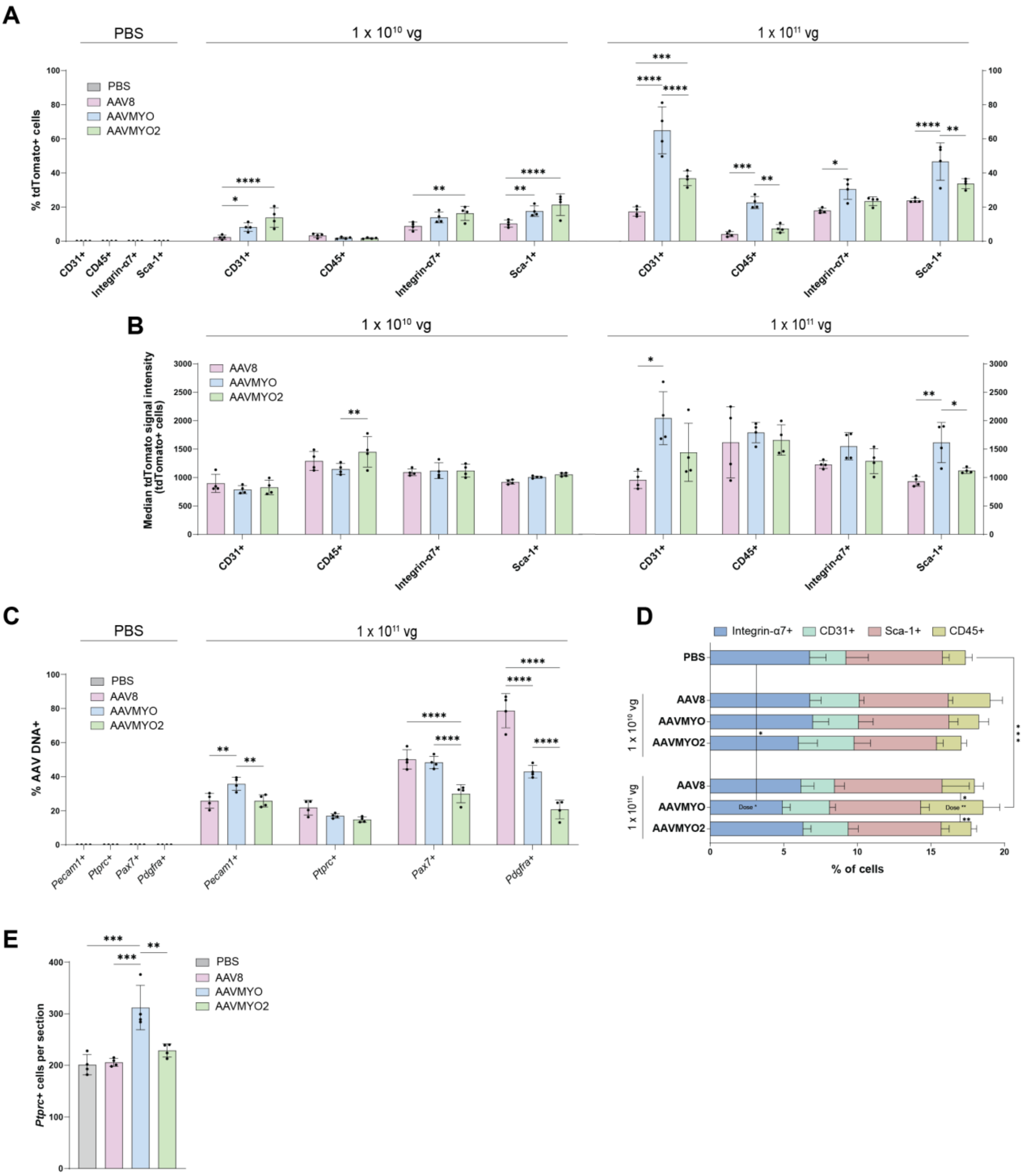
A
PBS
1 x 10¹⁰ vg
1 x 10¹¹ vg
% tdTomato+ cells
PBS
AAV8
AAVMYO
AAVMYO2
CD31+
CD45+
Integrin-α7+
Sca-1+
B
Median tdTomato signal intensity (tdTomato+ cells)
C
% AAV DNA+
Pecam1+
Ptprc+
Pax7+
Pdgfra+
D
% of cells
E
Ptprc+ cells per section

Fig. S5: Transduction rates and effect on muscle composition of IM-injected AAV8, AAVMYO and AAVMYO2. **A** Quantification of the proportion of tdTomato+ cells in CD31+, CD45+, Integrin-α7+ and Sca-1+ cells. **B** Quantification of the median tdTomato signal intensity in CD31+/tdTomato+, CD45+/tdTomato+, Integrin-α7+/tdTomato+ and Sca-1+/tdTomato+ cells. **C** Quantification of the proportion of cell types co-localizing with AAV DNA by smFISH. For each cell type, the proportion of AAV DNA+ cells was calculated by dividing the number of DAPI+ nuclei co-localizing with both AAV DNA and the cell-specific marker by the total number of DAPI+ nuclei co-localizing with the cell-specific marker. **D** Quantification of the proportion of CD31+, CD45+, Integrin-α7+ and Sca-1+ cells detected during flow cytometry. n = 4. **E** Quantification of the number of *Ptprc*-expressing immune cells per cross-section by smFISH. Data are shown as means ± SD. In **A**, **B** and **C**, statistics were evaluated using one-way ANOVAs with Tukey's multiple comparisons test. In **D**, statistics were evaluated using two-way ANOVAs with Tukey's multiple comparisons test and dose effects are shown as Dose* inside of the bar at the higher titer. In **E**, statistics were evaluated using an unpaired student's t-test. $p < 0.05$ = *, $p < 0.01$ = **, $p < 0.001$ = ***, $p < 0.001$ = ****.

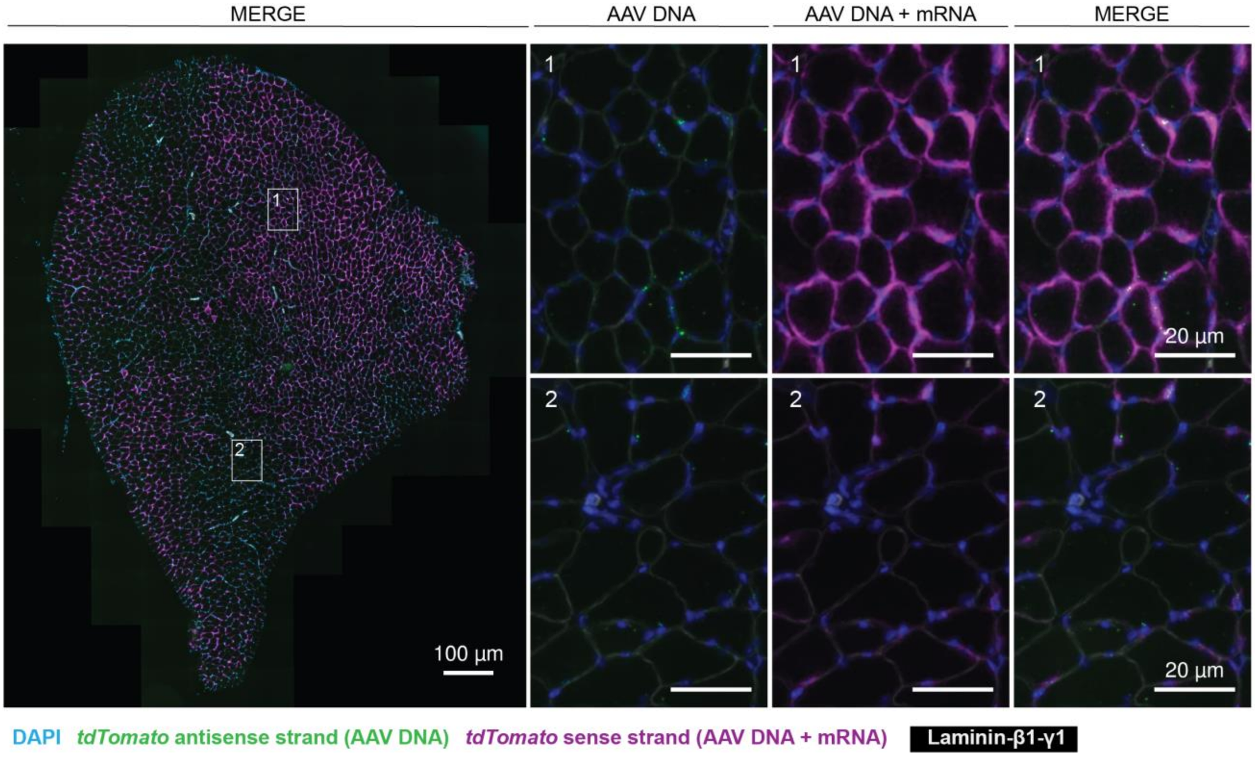

Fig. S6: Probes against *tdTomato* sense and antisense strands detect *tdTomato* DNA and mRNA post-AAV administration. smFISH of full TA section shows the presence of *tdTomato* sense (magenta) and antisense (green) strands in the muscle 3 weeks post-IM administration of 1 x 10^{10} vg per TA of AAVMYO. The probe for the *tdTomato* antisense strand is specific to AAV DNA, while the probe for the sense strand detects AAV DNA and mRNA. Box 1 highlights an area of high AAV transduction, box 2 shows an area of the same muscle with low transduction. Laminin-β1-γ1 is stained in white, DAPI in blue.

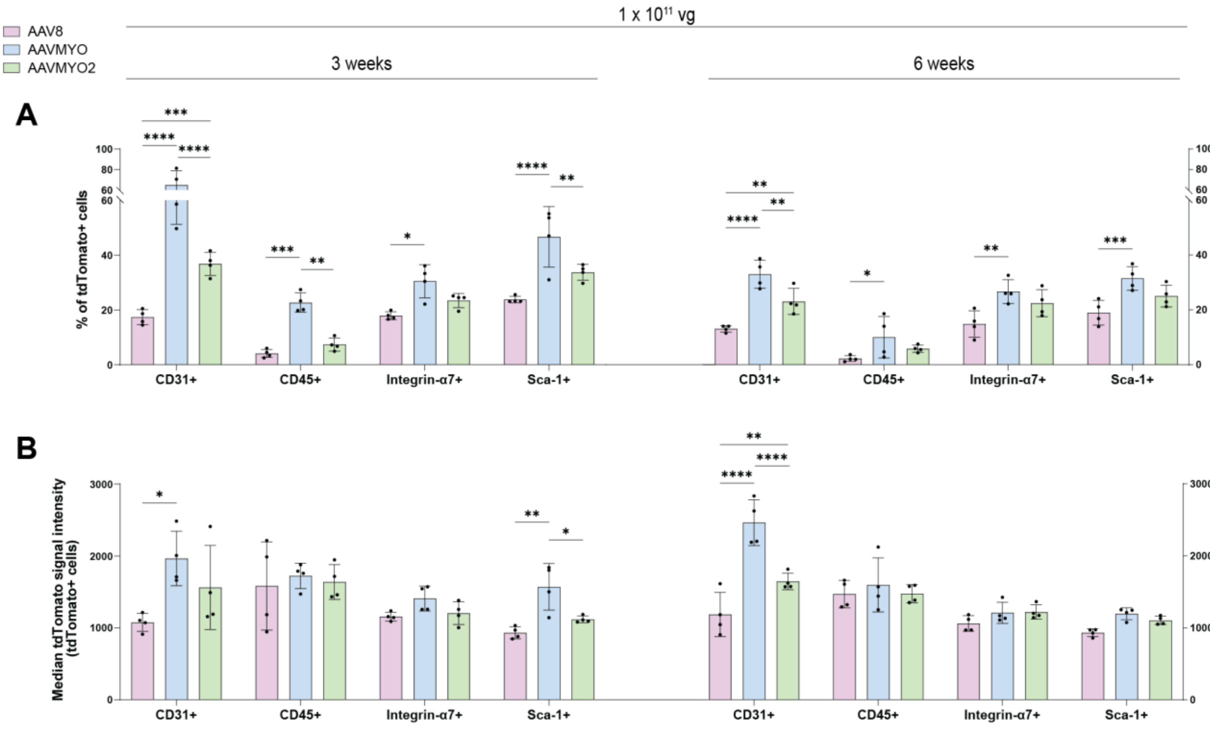

Fig. S7: Quantification of AAV8, AAVMYO and AAVMYO2 transduction levels 3 and 6 weeks post-IM administration. **A** Quantification of the proportion of tdTomato+ cells in CD31+, CD45+, Integrin-α7+ and Sca-1+ cells 3 and 6 weeks post-administration of 1 x 10^{11} vg per TA. **B** Quantification of the median tdTomato signal intensity in CD31+/tdTomato+, CD45+/tdTomato+, Integrin-α7+/tdTomato+ and Sca-1+/tdTomato+ cells 3 and 6-weeks post AAV-administration. Data are shown as means ± SD. Statistics were evaluated for each cell type using one-way ANOVAs with Tukey's multiple comparisons test. $p < 0.05$ = *, $p < 0.01$ = **, $p < 0.001$ = ***, $p < 0.001$ = ****.

<u>Table S1: Flow cytometry quantifications of the proportion of cell types and tdTomato+ cells after IV injection.</u>

	% of single cells				% tdTomato+			
Treatment	CD31	CD45	Integrin-a7	Sca-1	CD31	CD45	Integrin-a7	Sca-1
PBS	4.58	0.83	5.2	6.86	0	0	0	0
AAV8 3E+12 vg/kg	4.44	0.92	4.88	6.85	0	0	0.04	0.038
AAVMYO 3E+12 vg/kg	2.3	1.15	6.26	8.57	1.14	0.21	4.65	1.01
AAVMYO2 3E+12 vg/kg	1.93	1.23	6.69	7.58	0.038	0	0.18	0.02
PBS	2.75	1.65	6.67	9.24	0	0	0	0
AAV8 3E+12 vg/kg	4.24	1.47	8.69	8.06	0.013	0.038	0.045	0.014
AAVMYO 3E+12 vg/kg	4.58	1.64	7.56	9.79	0.21	0.062	1.64	0.53
AAVMYO2 3E+12 vg/kg	2.47	1.14	7.33	7.81	0.024	0.053	0.21	0.21
PBS	4.72	0.85	6.38	7	0	0	0	0
AAV8 3E+12 vg/kg	2.61	2.26	7.78	9.97	0.027	0	0.027	0.028
AAVMYO 3E+12 vg/kg	5.11	2.46	6.58	9.56	0.074	0.038	0.51	0.13
AAVMYO2 3E+12 vg/kg	2.8	1.34	8.35	9.27	0.042	0.088	0.063	0.057
PBS	1.24	1.36	6.63	8.77	0	0	0.013	0.019
AAV8 3E+12 vg/kg	3.08	2.43	5.59	7.84	0	0	0.037	5.32E-03
AAVMYO 3E+12 vg/kg	5.84	2.59	7.42	9.84	0.53	0.48	2.81	2.81
AAVMYO2 3E+12 vg/kg	5.45	1.3	5.79	8.65	0.032	0	0.25	0.22
AAV8 3E+13 vg/kg	2.73	1.22	6.27	7.78	0.22	0	1.02	0.21
AAVMYO 3E+13 vg/kg	2.67	1.01	6.25	7.34	5.95	2.53	21.3	21.4
AAVMYO2 3E+13 vg/kg	5.11	2.12	8.27	11.6	0.23	0.2	2.58	1.17
AAV8 3E+13 vg/kg	2.91	1.73	6.81	7.33	0.15	0	0.7	0.24
AAVMYO 3E+13 vg/kg	3.53	0.82	6.55	7.61	9.73	5.96	31.8	36.8
AAVMYO2 3E+13 vg/kg	4.13	1.76	7.72	8.49	1.23	0.46	3.99	5.2
AAV8 3E+13 vg/kg	5.84	2.16	8.25	7.8	0.057	0	0.72	0.22
AAVMYO 3E+13 vg/kg	4.65	1.16	5.96	6.62	4.64	2.64	16.3	18.6
AAVMYO2 3E+13 vg/kg	4.81	1.55	8.53	8.58	0.18	0.15	1.32	0.93
AAV8 3E+13 vg/kg	1.62	2	8.79	10.3	0.27	0	0.78	0.14
AAVMYO 3E+13 vg/kg	3.06	2.22	9.66	9.14	17.9	3.76	21	27.5
AAVMYO2 3E+13 vg/kg	2.38	1.29	9.61	10.4	0.52	0.34	1.94	3.2

Table S2: Flow cytometry quantifications of the median tdTomato signal and number of tdTomato+ cells after IV injection.

	Median tdTomato signal				Number of tdTomato+ cells			
Treatment	CD31	CD45	Integrin-a7	Sca-1	CD31	CD45	Integrin-a7	Sca-1
PBS	n/a	n/a	n/a	n/a	0	0	0	0
AAV8 3E+12 vg/kg	n/a	n/a	546	1373	0	0	3	4
AAVMYO 3E+12 vg/kg	738	500	793	674	43	4	475	141
AAVMYO2 3E+12 vg/kg	650	n/a	639	658	1	0	16	2
PBS	n/a	n/a	n/a	n/a	0	0	0	0
AAV8 3E+12 vg/kg	525	540	599	537	1	1	7	2
AAVMYO 3E+12 vg/kg	828	512	732	661	19	2	246	102
AAVMYO2 3E+12 vg/kg	16652	512	642	893	1	1	26	27
PBS	n/a	n/a	n/a	n/a	0	0	0	0
AAV8 3E+12 vg/kg	8794	n/a	571	801	1	0	3	4
AAVMYO 3E+12 vg/kg	611	527	699	708	8	2	72	26
AAVMYO2 3E+12 vg/kg	677	494	541	908	2	2	9	9
PBS	n/a	n/a	522	732	0	0	2	4
AAV8 3E+12 vg/kg	n/a	n/a	546	544	0	0	5	1
AAVMYO 3E+12 vg/kg	685	817	794	784	45	18	303	402
AAVMYO2 3E+12 vg/kg	630	n/a	690	665	3	0	25	32
AAV8 3E+13 vg/kg	567	n/a	789	796	8	0	86	22
AAVMYO 3E+13 vg/kg	1067	1278	1150	941	254	41	2133	2510
AAVMYO2 3E+13 vg/kg	650	755	781	708	22	8	397	252
AAV8 3E+13 vg/kg	507	n/a	679	525	1	0	11	4
AAVMYO 3E+13 vg/kg	840	1097	1320	1112	744	106	4517	6064
AAVMYO2 3E+13 vg/kg	740	905	830	793	108	17	652	935
AAV8 3E+13 vg/kg	929	n/a	720	760	5	0	89	26
AAVMYO 3E+13 vg/kg	747	1348	1026	976	316	45	1421	1808
AAVMYO2 3E+13 vg/kg	641	803	703	685	23	6	299	212
AAV8 3E+13 vg/kg	537	n/a	743	788	8	0	125	26
AAVMYO 3E+13 vg/kg	743	1376	1049	1038	790	120	2917	3611
AAVMYO2 3E+13 vg/kg	972	1145	720	798	17	6	257	460

Table S3: Flow cytometry quantifications of the proportion of cell types and tdTomato+ cells 3 weeks after IM injection.

	% of single cells				% tdTomato+			
Treatment	CD31	CD45	Integrin-a7	Sca-1	CD31	CD45	Integrin-a7	Sca-1
PBS	0.85	2.12	8.17	6.91	0	0	0	0
AAV8 1E+10 vg	3.24	2.47	7.6	5.93	0.78	4.28	9.88	11.1
AAVMYO 1E+10 vg	3.26	2.73	8.56	6.26	5.15	1.29	18.2	22
AAVMYO2 1E+10 vg	2.96	2.09	7.93	5.79	20.5	2.44	17.1	26
PBS	1.75	1.04	5.48	6.1	0	0	0	0
AAV8 1E+10 vg	3.35	2.51	7.11	6.18	3.9	3.84	11.2	12.7
AAVMYO 1E+10 vg	3.93	1.15	6.22	5.44	8.09	2.5	14.4	17.4
AAVMYO2 1E+10 vg	3.23	1.48	5.4	5.4	7.15	2.93	10.3	12
PBS	2.96	1.39	6.94	6.98	0	0	0	0
AAV8 1E+10 vg	3.05	4.09	6.59	6.39	2.12	3.36	5.69	7.92
AAVMYO 1E+10 vg	1.67	2	6.72	6.01	11.3	2.08	11.6	15.1
AAVMYO2 1E+10 vg	3.51	1.84	5.28	6.13	11.7	1.87	20	22.9
PBS	4.33	1.68	6.47	6.26	0	0	0	7.31E-03
AAV8 1E+10 vg	3.8	2.29	5.83	5.7	2.17	1.56	8.4	9.52
AAVMYO 1E+10 vg	3.55	2.25	6.43	6.91	8.19	1.39	11.3	15.8
AAVMYO2 1E+10 vg	5.41	1.31	5.42	5.14	15.8	2.97	17.8	24.5
AAV8 1E+11 vg	1.81	1.99	7.44	9.69	14.5	2.5	17.1	23.1
AAVMYO 1E+11 vg	3.24	4.95	5.28	6.76	70.5	27.4	36.1	47
AAVMYO2 1E+11 vg	2.49	1.75	6.54	6.31	36.3	6.75	24.5	36.5
AAV8 1E+11 vg	3.26	3.09	5.37	6.78	15.8	5.98	19.9	25.6
AAVMYO 1E+11 vg	3.39	4.32	5.44	6.6	81.2	20.1	33.3	55.1
AAVMYO2 1E+11 vg	3.65	2.22	5.36	6.03	41.6	4.88	24.5	35
AAV8 1E+11 vg	1.88	1.98	5.83	5.27	18.6	3.4	18	23.3
AAVMYO 1E+11 vg	2.65	2.63	4.56	5.89	49.7	19.8	22.1	31
AAVMYO2 1E+11 vg	2.49	2.47	5.53	7.07	31.4	7.23	25.1	33.7
AAV8 1E+11 vg	2.19	1.74	6.05	7.53	20.5	4.53	16.6	23.2
AAVMYO 1E+11 vg	3.55	5.08	4.38	5.52	58.6	23.3	30.4	53.6
AAVMYO2 1E+11 vg	3.65	1.74	5.04	5.87	38	10.6	19.5	29.7

Table S4: Flow cytometry quantifications of the median tdTomato signal and number of tdTomato+ cells 3 weeks after IM injection.

	Median tdTomato signal				Number of tdTomato+ cells			
Treatment	CD31	CD45	Integrin-a7	Sca-1	CD31	CD45	Integrin-a7	Sca-1
PBS	n/a	n/a	n/a	n/a	0	0	0	0
AAV8 1E+10 vg	1136	1368	1177	901	41	171	1216	1068
AAVMYO 1E+10 vg	807	1051	1320	1032	204	43	1900	1680
AAVMYO2 1E+10 vg	862	1123	1246	1018	1231	103	2742	3048
PBS	n/a	n/a	n/a	n/a	0	0	0	0
AAV8 1E+10 vg	805	1489	1069	946	246	182	1505	1481
AAVMYO 1E+10 vg	737	1270	1108	1010	730	66	2062	2179
AAVMYO2 1E+10 vg	740	1484	1065	1032	351	66	841	979
PBS	n/a	n/a	n/a	n/a	0	0	0	0
AAV8 1E+10 vg	846	1179	1084	879	132	281	765	1033
AAVMYO 1E+10 vg	879	1177	1046	1004	456	101	1898	2206
AAVMYO2 1E+10 vg	994	1421	1177	1078	883	74	2264	3014
PBS	n/a	n/a	n/a	738	0	0	0	1
AAV8 1E+10 vg	807	1127	1036	964	148	64	881	976
AAVMYO 1E+10 vg	727	1101	1006	984	476	51	1187	1786
AAVMYO2 1E+10 vg	713	1777	996	1080	2005	91	2257	2945
AAV8 1E+11 vg	1072	945	1235	846	515	97	2490	4389
AAVMYO 1E+11 vg	2006	1758	1544	1501	5933	3521	4948	8255
AAVMYO2 1E+11 vg	2410	1432	1381	1093	1572	206	2792	4014
AAV8 1E+11 vg	912	1987	1143	880	835	299	1728	2803
AAVMYO 1E+11 vg	2485	1877	1570	1801	7714	2435	5073	10196
AAVMYO2 1E+11 vg	1190	1459	1172	1186	2957	211	2562	4109
AAV8 1E+11 vg	1104	1183	1156	1032	1055	204	3185	3717
AAVMYO 1E+11 vg	1709	1470	1258	1141	3719	1471	2849	5164
AAVMYO2 1E+11 vg	1489	1946	1256	1112	1879	429	3333	5730
AAV8 1E+11 vg	1213	2214	1089	968	1071	189	2402	4177
AAVMYO 1E+11 vg	1661	1788	1256	1839	8286	4714	5305	11795
AAVMYO2 1E+11 vg	1156	1715	1004	1082	3294	437	2330	4144

Table S5: Flow cytometry quantifications of the proportion of cell types and tdTomato+ cells 6 weeks after IM injection.

	% of single cells				% tdTomato+			
Treatment	CD31	CD45	Integrin-a7	Sca-1	CD31	CD45	Integrin-a7	Sca-1
AAV8 1E+11 vg	1.34	2.8	7.18	9.73	14	1.1	20.6	24.1
AAVMYO 1E+11 vg	1.61	3.1	7.05	7.92	38.7	4.69	32.6	36.8
AAVMYO2 1E+11 vg	2.58	2.36	7.15	8.07	29.7	7.5	28.8	30.3
AAV8 1E+11 vg	1.54	3.02	8.37	8.05	14	3.7	15.9	21.1
AAVMYO 1E+11 vg	1.05	1.48	6.15	6.37	27.7	2.77	26	27.1
AAVMYO2 1E+11 vg	1.08	1.21	6.33	6.15	22.3	5.5	23.7	25.8
AAV8 1E+11 vg	1.05	1.6	4.8	4.57	11.5	2.05	9	13.9
AAVMYO 1E+11 vg	2.1	4.77	6.76	6.9	29.8	18.6	22.2	28.9
AAVMYO2 1E+11 vg	1.88	3.2	8.47	11.2	18.5	4.35	19.3	21.2
AAV8 1E+11 vg	1.83	2.97	5.76	6.28	12.9	1.99	13.9	16.8
AAVMYO 1E+11 vg	1.34	1.93	5.75	7.78	35.7	14	25.9	33.1
AAVMYO2 1E+11 vg	2.85	1.62	8.7	8.07	21.8	5.94	17.9	22.9

Table S6: Flow cytometry quantifications of the median tdTomato signal and number of tdTomato+ cells 6 weeks after IM injection

	Median tdTomato signal				Number of tdTomato+ cells			
Treatment	CD31	CD45	Integrin-a7	Sca-1	CD31	CD45	Integrin-a7	Sca-1
AAV8 1E+11 vg	1612	1290	1183	970	437	72	3444	5457
AAVMYO 1E+11 vg	2832	2123	1424	1213	1208	282	4458	5665
AAVMYO2 1E+11 vg	1815	1576	1363	1172	945	218	2537	3014
AAV8 1E+11 vg	1183	1320	1117	972	394	203	2419	3095
AAVMYO 1E+11 vg	2189	1251	1132	1074	653	92	3595	3872
AAVMYO2 1E+11 vg	1570	1591	1172	1059	534	147	3318	3500
AAV8 1E+11 vg	903	1606	968	858	274	74	974	1433
AAVMYO 1E+11 vg	2624	1440	1108	1244	1004	1421	2412	3203
AAVMYO2 1E+11 vg	1621	1384	1202	1132	1049	419	4928	7150
AAV8 1E+11 vg	1044	1661	970	927	392	98	1327	1746
AAVMYO 1E+11 vg	2202	1570	1168	1251	1226	690	3813	6596
AAVMYO2 1E+11 vg	1573	1350	1141	1051	1107	171	2765	3288

Table S7: List of antibodies used.

Antibody	Company	Reference	Dilution
Rat anti-CD31, Alexa Fluor 700 conjugated	BioLegend (San Diego, California)	102444	1/200
Rat anti-CD45, Alexa Fluor 488 conjugated	BioLegend (San Diego, California)	103122	1/400
Rat anti-Integrin-α7, Alexa Fluor 647 conjugated	Ablab (Sao Paulo, Brazil)	67-0010-05	1/1000
Rat anti-Sca-1, Bright Violet 421 conjugated	BioLegend (San Diego, California)	108128	1/400
Anti-Laminin-β1-γ1 produced in rabbit	Sigma-Aldrich (St. Louis, Missouri)	L9393	1/100
Goat anti-Rabbit IgG (H+L) Cross-Absorbed Secondary Antibody, Alexa Fluor 750	Thermo Fisher Scientific (Waltham, Massachusetts)	A-21039	1/100

Table S8: List of RNAscope probes used.

Probe	Channel	Catalog Number
tdTomato antisense (to detect sense sequence)	C1	317041
Probe diluent	C1	300041
tdTomato sense (to detect antisense sequence)	C2	539641-C2
Pdgfra	C3	480661-C3
Pecam1	C3	316721-C3
Ptprc	C3	318651-C3
Pax7	C3	314181-C3
