# Supplementary material for: AAV capsids target muscle-resident cells with different efficiencies—A comparative study between AAV8, AAVMYO, and AAVMYO2
Source: Mol Ther Methods Clin Dev. 2025 Mar 14;33(2):101451. doi: 10.1016/j.omtm.2025.101451 (PMC11987650; doi:10.1016/j.omtm.2025.101451)
Supplement: Document S2. Article plus supplemental information [file mmc2.pdf]

Molecular Therapy
Methods & Clinical Development
Original Article

AAV capsids target muscle-resident cells with different efficiencies—A comparative study between AAV8, AAVMYO, and AAVMYO2

Timothy J. McGowan,[1] Nicolas Lewerenz,[1] Eleonora Maino,[1] Marco Thürkauf,[1] Lena Jörin,[1] and Markus A. Rüegg[1]

[1]Biozentrum, University of Basel, Spitalstrasse 41, 4056 Basel, Switzerland

Adeno-associated viruses (AAVs) of different serotypes are commonly used in gene therapies and gene interrogation studies to deliver transgenes to skeletal muscle in humans and mice. While efficient muscle fiber transduction is possible, little is known of their capacity to transduce muscle-residing mononuclear cells. Here, we addressed this question for AAV8 and the two myotropic AAVs, AAVMYO and AAVMYO2, by engineering them to express the tdTomato gene. AAVs were then injected intramuscularly or intravenously at two different doses into adult mice followed by flow-cytometry-based isolation of endothelial cells, immune cells, muscle stem cells, and fibro-adipogenic progenitor cells from the *tibialis anterior* muscle. Overall, we noted varying rates of tdTomato expression across all cell types. Transduction efficiency fluctuated in AAV serotype-dependent, titer-dependent, administration-dependent, and cell-dependent manners. By visualizing AAV DNA *in vivo*, we confirmed that AAV8, AAVMYO, and AAVMYO2 deliver transgenes to muscle-residing mononuclear cells. We show that mononuclear cells are also successfully transduced in the dy^W/dy^W mouse model of LAMA2-related muscular dystrophy. Altogether, we demonstrate that muscle-residing mononuclear cells are transduced by AAVs and provide an insightful guidance for researchers aiming to target muscle-resident mononuclear cells in their studies.

INTRODUCTION

Skeletal muscle fibers are convenient targets for gene therapies and gene interrogation studies due to their low turnover and their high protein production.[1] The current leading platform for gene delivery in skeletal muscles are recombinant adeno-associated viruses (AAVs). Various AAV serotypes have thus far been tested for gene delivery to muscle fibers. AAV1,[2,3] AAV2,[4] and AAV9[5] have been used to knockdown and overexpress genes of interest in murine skeletal muscle. In a clinical context, AAVrh74 is used in the first gene therapy approved for Duchenne muscular dystrophy (DMD); Food and Drug Administration submission tracking number: 125781. AAV9 and AAV8 are also being investigated in ongoing clinical trials for DMD (NCT03362502; NCT05693142), X-linked myotubular myopathy (NCT03199469) and FKRP-related limb-girdle muscular dystrophy 9 (LGMDR9; NCT05224505).

Recent engineering of AAV capsids has resulted in the isolation of viruses with improved targeting of skeletal muscle compared with AAV8 or AAV9.[6–8] These next-generation AAVs have been used in mice and non-human primates and might be amenable to future human gene therapies that tackle neuromuscular diseases. Additionally, we have recently shown that AAVMYO is highly efficient in delivering single guide RNAs to mice that express Cas9 in skeletal muscle fibers thereby enabling somatic, muscle fiber-specific gene knockouts.[9] While comparative analyses of muscle fiber transduction rates by different vectors have been performed, it remains unclear whether other muscle-resident cells are being transduced and to what extent.

Skeletal muscles are heterogeneous tissues composed of polynucleated muscle fibers and mononuclear cells, which contribute to approximately half of the tissues' nuclei.[10,11] Among these mononuclear cells are endothelial cells, immune cells, muscle satellite/stem cells (MuSCs), and fibro-adipogenic progenitor cells (FAPs) that have all been demonstrated to play an important role in the maintenance of tissue homeostasis.[12–16] Due to their implication in numerous muscular diseases, these cells are important therapeutic targets. In DMD and other muscular dystrophies, defective muscle regeneration and/or MuSC function contribute to the disease phenotypes.[12,17–19] Similarly, FAPs have been implicated in DMD disease progression and have been described as potential therapeutic targets to reduce disease-associated fibrosis.[20]

To develop gene therapies targeting these muscle-residing mononuclear cells, it must be determined if they can be transduced by AAVs. This is also important information for gene interrogation studies, as the combination of ubiquitous promoters and transduction by AAVs may lead to transgene expression in these mononuclear cells. Consequently, the function of these cells can be impacted, provoking changes in the tissue that are wrongfully attributed to transgene expression in the muscle fibers. It is therefore important to determine if mononuclear cells are being transduced by AAVs and

Received 21 September 2024; accepted 11 March 2025;
https://doi.org/10.1016/j.omtm.2025.101451.

Correspondence: Markus A. Rüegg, Biozentrum, University of Basel, Spitalstrasse 41, 4056 Basel, Switzerland.
E-mail: markus-a.ruegg@unibas.ch

© 2025 The Authors. Published by Elsevier Inc. on behalf of The American Society of Gene and Cell Therapy.
This is an open access article under the CC BY license (http://creativecommons.org/licenses/by/4.0/).

if so, how much is achieved by the different serotypes, at different titers, and *via* different routes of administration.

In the current study, we used flow cytometry to quantify the transduction rates of CD31+ endothelial cells, CD45+ immune cells, Integrin-α7+ MuSCs, and Sca-1+ FAPs in the *tibialis anterior* (TA) of adult C57BL/6 mice injected intravenously (i.v.) or intramuscularly (IM) with AAV8, AAVMYO, or AAVMYO2 at two different concentrations. Our results demonstrate that all AAVs are able to transduce endothelial cells, immune cells, MuSCs, and FAPs with surprisingly little difference between the AAVs at low concentrations while at a 10-fold higher concentration, AAVMYO outperformed the other AAVs. Using single molecule fluorescence *in situ* hybridization (smFISH), we confirmed that viral DNA encoding tdTomato (herein called AAV DNA) was delivered to endothelial cells, immune cells, MuSCs, and FAPs *in vivo*. We also show that tdTomato expression persists in the cells for 6 weeks post-AAV administration, with only a small decrease in expression observed from 3 to 6 weeks post-injection. Finally, we show that intramuscularly injected AAVMYO delivers transgenes to muscle-residing mononuclear cells in the dy^W/dy^W mouse model of LAMA2-related muscular dystrophy, demonstrating that these cells can be targeted to express therapeutic transgenes in the context of muscular diseases. All in all, our work offers guidance for researchers developing gene therapies and interrogating gene function via AAVs in skeletal muscle by pointing out the dose- and injection-specific differences among AAV8, AAVMYO, and AAVMYO2.

RESULTS

Characterization of mononuclear cell transduction after intravenous injection of AAV8, AAVMYO, and AAVMYO2

AAV8, AAVMYO, and AAVMYO2 transduce skeletal muscle after i.v. administration in adult mice.[6,7] While histological analyses show strong expression of the transgene in the muscle fibers,[6,7] it is unclear whether the mononuclear cells present in the skeletal muscle are also transduced. To address this, we generated AAV8, AAVMYO, and AAVMYO2 that contained the same transgene cassette encoding tdTomato under the control of the CMV promoter. Viruses were then injected into the tail vein of 8- to 10-week-old male C57BL/6 mice at a low (3.0×10^{12} vector genomes/kilogram [vg/kg]) and a high concentration (3.0×10^{13} vg/kg). Three weeks post-injection, a time point used by others to quantify and compare the levels of AAV-mediated transgene expression in skeletal muscle,[7,21] we collected TA muscles and generated a single-cell suspension for staining with cell type-specific markers CD31, CD45, Integrin-α7, and Sca-1. These are well-described cell surface proteins that have been used consistently to isolate endothelial cells, immune cells, MuSCs, and FAPs from skeletal muscle,[22–26] respectively. We then used flow cytometry to determine the proportion of tdTomato+ cells within these cell types (see experimental outline in Figure 1A; flow cytometry panel in Figure S1).

At the low concentration, only a few tdTomato+ cells were detected (Figures 1B–1D, S2, and S3A). For all cell types, the highest number of tdTomato+ cells were seen after AAVMYO administration, followed by AAVMYO2 and AAV8, although the difference between the serotypes only became significant in MuSCs and FAPs (Figure S3A). In AAVMYO-injected mice, 2.40% of the MuSCs expressed tdTomato vs. 0.18% for AAVMYO2 and 0.04% for AAV8 (Figures 1B–1D). With FAPs, AAVMYO led to tdTomato expression in 1.12% of the cells, vs. 0.13% for AAVMYO2 and 0.02% for AAV8 (Figures 1B–1D). We also quantified the median tdTomato signal emitted by tdTomato-expressing cells, as it may be influenced by the number of vector genomes per cell.[7,27] Upon quantification, the median tdTomato signal emitted by tdTomato+ cells (only samples with >5 tdTomato+ cells were included) was not significantly different between serotypes (Figure S3B), suggesting that the per-cell transduction was similar for all AAV serotypes.

With a 10-fold increase in the amount of virus to 3.0×10^{13} vg/kg, more tdTomato+ cells were detected (Figures 1B–1D, S2, and S3A). With the exception of immune cells, AAVMYO led to significantly higher proportions of tdTomato+ cells than AAV8 and AAVMYO2 (Figure S3A). This difference was particularly striking in MuSCs and FAPs (Figure S3A). In these two cell types, tdTomato expression was detected in more than 20% of the cells after injection of AAVMYO (Figure 1C, upper panel). AAVMYO was also superior in the median tdTomato signal of transduced cells compared with AAV8 and AAVMYO2-injected mice (Figure S3B). All in all, these data clearly show that AAVMYO is superior in promoting transgene expression in mononuclear cells in skeletal muscle.

After comparing the effects of the different AAV serotypes on a given cell population, we next examined whether a particular serotype showed relative preference for a particular cell type. AAV8 at the high dose showed some preference for MuSCs compared with endothelial cells and FAPs while transgene expression was undetected in immune cells (Figure 1B, upper panel). In contrast, both AAVMYO and AAVMYO2 transduced FAPs with similar efficacy as MuSCs (Figures 1C and 1D, upper panels). Thus, the two engineered AAVMYO variants[6,7] achieve transgene expression in FAPs and MuSCs equally well while the naturally occurring AAV8 serotype has a preference toward MuSCs. As noted above, AAVMYO led to tdTomato expression in more than 20% of MuSCs and FAPs, while with AAVMYO2 this number dropped to approximately 2.5% and in the case of AAV8 to less than 1% in MuSCs (Figures 1B–1D, upper panels). When comparing tdTomato expression between the two doses, the percentage of tdTomato-expressing cells increased between 10- and 20-fold irrespective of the AAV serotype (Figures 1B–1D, upper panels). Hence, increasing the dose improves the transduction rate linearly, indicating that none of the AAV serotypes reached saturation. Interestingly, with AAV8 and AAVMYO2, the rather big increase in the proportion of tdTomato-expressing cells did not result in a big difference in the expression levels of the tdTomato transgene (Figures 1B and 1D; lower panels). Only the tdTomato-expressing immune cells, MuSCs, and FAPs of AAVMYO-treated mice saw a dose-dependent increase in tdTomato signal (Figure 1C, lower panel).

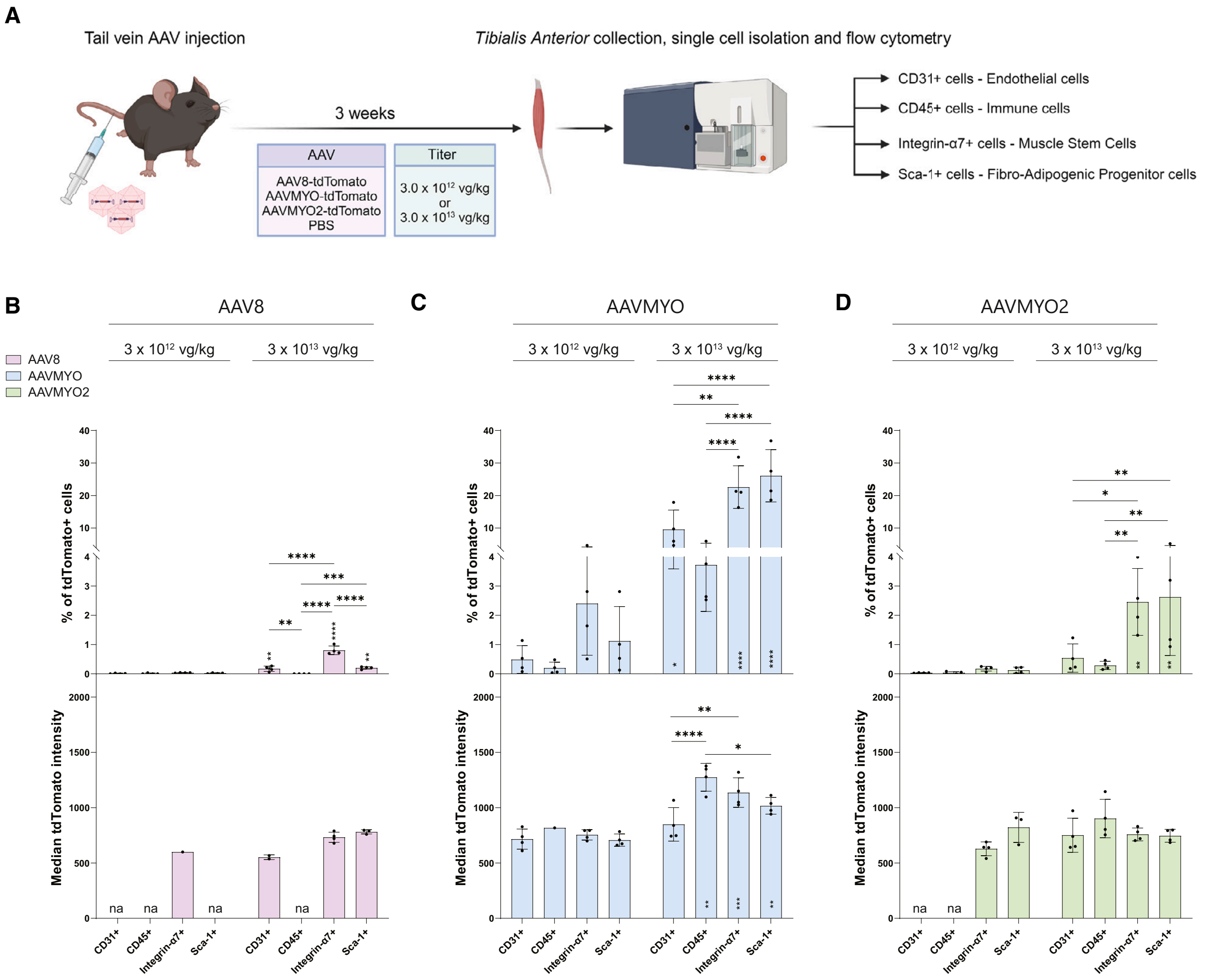

Figure 1. Transduction of mononuclear cells in the TA after i.v. injection of AAV8, AAVMYO, and AAVMYO2

(A) Schematic representation of the experimental procedure. 8- to 10-week-old male adult C57BL/6 were injected i.v. with PBS, AAV8, AAVMYO, or AAVMYO2 at the titer of 3.0×10^{12} or 3.0×10^{13} vg/kg ($n = 4$ mice per condition). All viruses contained a tdTomato transgene driven by a CMV promoter to label transduced cells. Three weeks post-injection, TAs were collected and digested to generate a single-cell suspension that was stained for CD31, CD45, Integrin-α7, and Sca-1. Flow cytometry was then used to characterize the proportion of tdTomato-expressing cells and the median tdTomato signal for each mouse. (B, C, and D) AAV-specific quantifications of the proportion of tdTomato+ cells (upper panels) and median tdTomato signal intensity (lower panels) in mice treated with AAV8 (B), AAVMYO (C), and AAVMYO2 (D) at 3.0×10^{12} or 3.0×10^{13} vg per kg. na = not applicable, when <5 tdTomato+ cells were detected in the cell population the median tdTomato signal was not measured. Data are shown as means ± SD. In (B), (C), and (D), statistics were evaluated using two-way ANOVAs with Tukey's multiple comparisons test and dose effects are shown by * within (C and D) or just above the bars (B) at the higher titer. *$p < 0.05$, **$p < 0.01$, ***$p < 0.001$, ****$p < 0.001$. Experimental scheme A was created with BioRender.com.

To investigate whether transduction of mononuclear cells by AAVs would eventually shift the proportions of the different cell types, we next determined their relative proportion irrespective of whether they were tdTomato-positive or not. None of the treatments caused a significant shift in the proportions of these cells (Figure S3C). These results suggest that the different AAVs tested did not disrupt the TAs' homeostatic composition of endothelial cells, immune cells, MuSCs, or FAPs. Altogether, AAVMYO showed the highest transduction of mononuclear cells after i.v. administration. For most cell populations and most AAV serotypes, the increase in the amount of virus injected also led to an increase in the number of tdTomato-expressing cells. At the low titer, MuSCs had the highest proportion of tdTomato+ cells irrespective of the AAV serotype. With the high titer, MuSCs and FAPs were similarly transduced in AAVMYO- and AAVMYO2-treated mice, but MuSCs remained the cell type with the highest number of tdTomato-expressing cells for AAV8-injected mice. When comparing the expression levels achieved for each cell type by the different AAV serotypes, we detected patterns that were serotype-specific. For example, AAV8 and AAVMYO both achieved transduction of MuSCs, but only

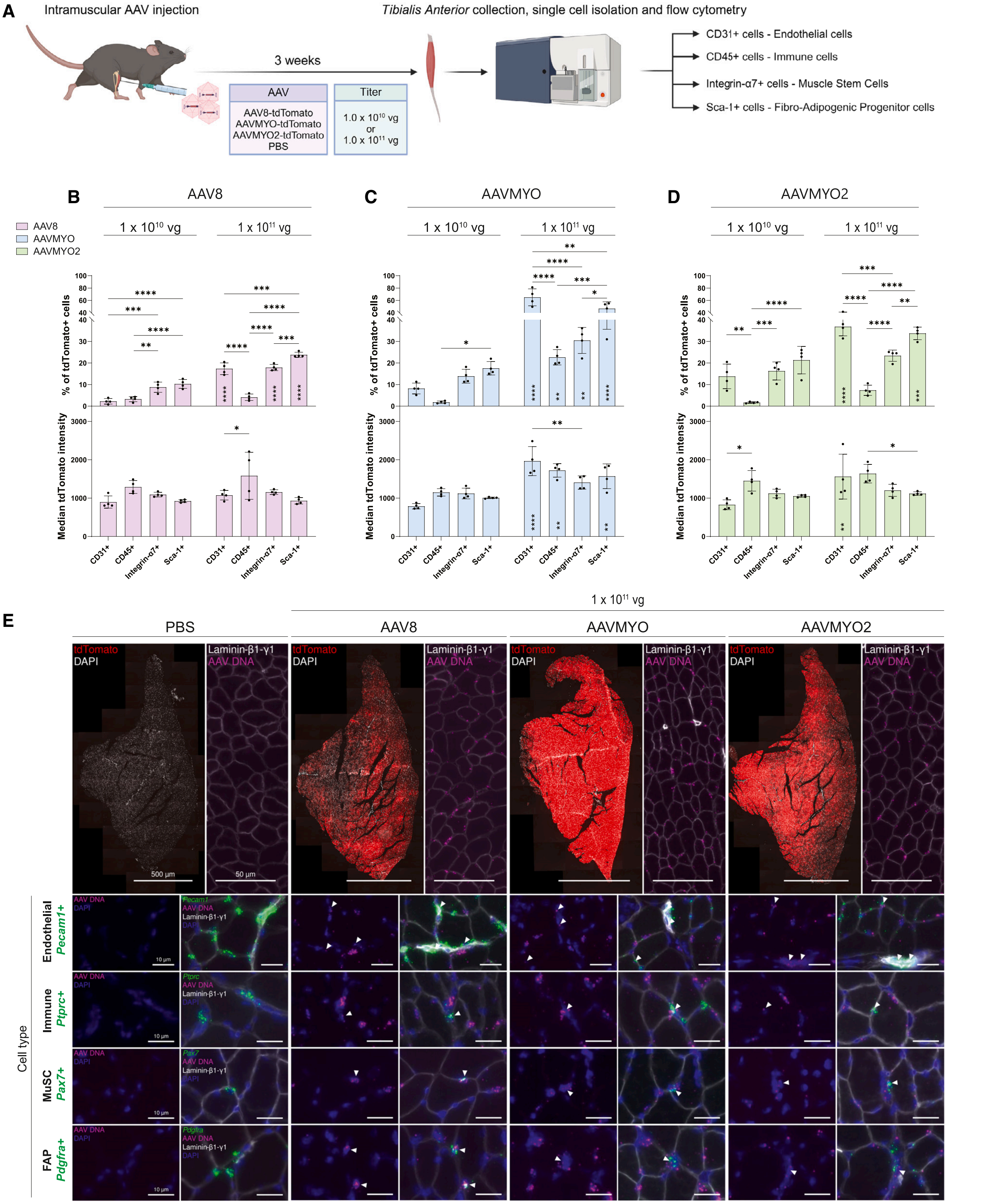

(legend on next page)

AAVMYO led to tdTomato expression in CD45+ cells (Figures 1B and 1C).

IM injection boosts the transduction of mononuclear cells in the TA

AAVs that are injected i.v. are delivered to skeletal muscle via the vascular system. One of the challenges with this route is the strong transduction of the liver by many AAVs. Hence, the number of AAV particles reaching muscle can be quite low. A strong retention of AAVs in the liver has been noted for AAV8 and AAVMYO,[7] while AAVMYO2 was specifically engineered to avoid the liver.[7] IM injection is therefore a means to determine transduction efficiency of the different AAV serotypes without this confounding factor. Furthermore, this application modality is often used to modulate gene expression in mouse muscle fibers.[2–5] To test transduction efficiency by local application, we injected 10-week-old male C57BL/6 mice IM with PBS, AAV8, AAVMYO, or AAVMYO2 with 1.0×10^{10} and 1.0×10^{11} vg per TA (equivalent to approximately 3.3×10^{11} vg/kg and 3.3×10^{12} vg/kg, respectively) (Figure 2A). Three weeks post-injection, TAs were collected and mononucleated cells were isolated as described above. At both concentrations, tdTomato+ cells were detected in all four cell types irrespective of the AAV serotypes (Figures 2B–2D, S4, and S5A). With the lower amount of AAVs, the most effective serotype was AAVMYO2, followed by AAVMYO, followed by AAV8 (Figure S5A). In endothelial cells and FAPs, the number of tdTomato-expressing cells obtained with AAVMYO and AAVMYO2 was significantly higher than with AAV8, while in immune cells, no significant differences were observed between the serotypes (Figure S5A). Despite AAVMYO's well-described efficiency in skeletal muscle,[6,9] no significant differences in the proportion of tdTomato-expressing MuSCs were observed between AAV8 and AAVMYO-treated mice (Figure S5A).

With a 10-fold increase in the number of vector genomes injected per TA, the percentage of cells expressing tdTomato was strongly increased in all cell types irrespective of the AAV serotype (Figures 2B–2D, S4, and S5A). Interestingly, the increase was usually less than 10-fold, suggesting saturation of the system. With the high dose, AAVMYO led to the highest number of tdTomato+ cells in all cell types, which is different from the low dose where this was achieved with AAVMYO2 (Figure S5A). In addition, at the high dose, most of the differences observed between AAVMYO2 and AAV8 at the low dose disappeared. The only remaining difference was the proportion of tdTomato-expressing CD31+ cells, which was significantly higher in the AAVMYO2-injected mice (Figure S5A). All in all, these data show that IM injection of AAVs, irrespective of the serotype, is much more efficient than i.v. injection in achieving transgene expression by mononuclear cells in skeletal muscle. At the high dose, AAVMYO is superior to the other AAVs, reaching tdTomato expression in 20% (immune cells) to 65% (endothelial cells) of the cells (Figure 2C, upper panel). At this high dose, all the AAVs led to tdTomato expression in 18%–31% of MuSCs (Figures 2B–2D, upper panels), which is an important finding for the treatment of muscular dystrophies using AAV-based gene therapy.

To get an estimate of whether increasing the dose would also improve transgene expression per cell, we measured tdTomato fluorescence intensity in the tdTomato+ cells. Overall, there were few differences in the median tdTomato signal emitted by tdTomato-expressing cells (Figures 2B–2D and S5B). At the low dose, only CD45+ cells transduced by AAVMYO2 showed a higher tdTomato signal than those transduced by AAVMYO (Figure S5B). At the high dose, the only differences in tdTomato signal came from Sca-1+ cells, where AAVMYO led to a stronger signal than AAV8 and AAVMYO2, and from CD31+ cells, where AAVMYO caused higher signal than AAV8 (Figure S5B).

When we focused on cell type transduction patterns, we observed that with 1.0×10^{10} vg per TA, some patterns were common to all AAVs. For example, all serotypes showed significantly higher tdTomato expression in Sca-1+ cells compared with CD45+ cells (Figures 2B–2D, upper panels). While the proportion of tdTomato-expressing cells showed important serotype-dependent fluctuations, the median tdTomato signal detected in the transduced cells was largely the same across cell types (Figures 2B–2D, lower panels). The only significant disparity was observed in AAVMYO2-treated mice, which had a higher signal in CD45+ cells compared with CD31+ cells (Figure 2D, lower panel).

Comparing the transduction rates for all cell types by each AAV showed that some of the transduction patterns described at the low

Figure 2. Transduction of mononuclear cells in the TA after IM injection of AAV8, AAVMYO and AAVMYO2

(A) Schematic representation of the experimental procedure. Ten-week-old male adult C57BL/6 mice were injected IM with PBS, AAV8, AAVMYO, or AAVMYO2 at the titer of 1.0×10^{10} (equivalent to approximately 3.3×10^{11} vg/kg) or 1.0×10^{11} vg per TA (equivalent to approximately 3.3×10^{12} vg/kg) (n = 4 mice per condition). All viruses contained a tdTomato transgene driven by a CMV promoter to allow the labeling of transduced cells with tdTomato. Three weeks post-injection, TAs were collected and digested to generate a single-cell suspension that was stained for CD31, CD45, Integrin-α7, and Sca-1. Flow cytometry was then used to characterize the proportion of tdTomato-expressing cells and the median tdTomato signal for each mouse. (B, C, and D) AAV-specific quantifications of the proportion of tdTomato+ cells and median tdTomato signal intensity in mice treated with AAV8 (B), AAVMYO (C), and AAVMYO2 (D) at 1.0×10^{10} or 1.0×10^{11} vg per TA. (E) Mid-belly cross-sections of TAs from mice injected IM with PBS or 1.0×10^{11} vg (equivalent to approximately 3.3×10^{12} vg/kg) of AAV8, AAVMYO, or AAVMYO2. Upper panels, left: overview of staining for tdTomato (red) and DAPI (white) in each condition. Upper panels, right: smFISH to detect *tdTomato* antisense strand (AAV DNA, magenta) and immunostaining for Laminin-β1-γ1 (white). Lower panels (cell type): smFISH to detect *tdTomato* antisense strand (AAV DNA) and mRNA of cell markers for endothelial cells (*Pecam1*), immune cells (*Ptprc*), MuSCs (*Pax7*), and FAPs (*Pdgfra*). DAPI (blue) was used to outline nuclei. Arrowheads indicate nuclei that are positive for the cell marker and viral DNA, demonstrating successful transduction. Data in (B)–(D) are shown as means ± SD. Statistical significance was determined by two-way ANOVAs with Tukey's multiple comparisons test and dose effects are shown by * within the bars at the higher titer. *$p < 0.05$, **$p < 0.01$, ***$p < 0.001$, ****$p < 0.001$. Experimental scheme (A) was created with BioRender.com.

dose persisted with the high dose. For example, all serotypes showed higher tdTomato expression in Sca-1+ cells compared with CD45+ cells (Figures 2B–2D, upper panels). There were also AAV-specific patterns detected after the injection of 1.0×10^{10} vg per TA that did not persist with the higher titer. This is the case in AAV8-injected mice, where CD31+ cells no longer had fewer tdTomato-expressing cells than Integrin-α7+ and Sca-1+ cells (17.4% vs. 17.9% and 23.8%, respectively) (Figure 2B, upper panel). Finally, we also examined whether the high dose of AAV would affect tdTomato expression in a specific cell type and whether this would be different between the serotypes. With all the serotypes, most of the cell types had more tdTomato-expressing cells at the high dose (Figures 2B–2D, upper panels). Notable exceptions were CD45+ immune cells with AAV8 (Figure 2B, upper panel) and CD45+ immune cells and Integrin-α7+ MuSCs with AAVMYO2 (Figure 2D, upper panel). When we compared the median tdTomato signal between low and high dose, there was no difference in AAV8-injected TA muscle irrespective of the cell type (Figure 2B, lower panel). In stark contrast, all cell types except the MuSCs expressed significantly higher levels of tdTomato when transduced with AAVMYO at the high dose (Figure 2C, lower panel). With AAVMYO2, significance between low and high dose was only reached in CD31+ endothelial cells (Figure 2D, lower panel).

To confirm our flow cytometry results demonstrating that AAVs deliver transgenes to muscle-residing mononuclear cells *in vivo*, we wanted to visualize the tdTomato transgene in muscle cross-sections to determine whether it co-localizes with the nuclei of mononuclear cells. For this, we used an smFISH probe that targets the *tdTomato* antisense strand and thereby enables visualization of the single-stranded and double-stranded AAV genome, but not the *tdTomato* mRNA (this probe is hereafter referred to as AAV DNA). We tested this probe in combination with a probe that detects the *tdTomato* sense strand, thereby recognizing the single-stranded AAV genome, the double-stranded AAV genome, and *tdTomato* mRNA (hereafter referred to as AAV DNA + mRNA).[28] The probe against AAV DNA + mRNA led to abundant signal in the cytosol and nuclei, whereas most of the AAV DNA probe was located in nuclei 3 weeks post-injection (Figure S6). Regions lacking AAV DNA + mRNA signal also lacked AAV DNA signal (Figure S6), confirming the specificity of the AAV DNA probe, which was subsequently used to detect the presence of AAV-delivered transgene in muscle-residing mononuclear cells.

In PBS-injected TA, both tdTomato protein and AAV DNA could not be detected, while TAs of mice that were injected with AAV8, AAVMYO, or AAVMYO2 were all strongly positive for both (Figure 2E). Although AAVMYO clearly led to the highest abundance of tdTomato protein (Figure 2E), there were no obvious differences in the number of AAV DNA-positive nuclei in AAV8, AAVMYO, and AAVMYO2-injected TAs (Figure 2E).

To visualize whether transgenes were delivered to muscle-residing mononuclear cells, we combined probes for AAV DNA with cell-specific mRNA markers: *Pecam1*, coding for CD31, for endothelial cells; *Ptprc*, coding for CD45, for immune cells; *Pax7* for MuSCs; and *Pdgfra* for FAPs. With this approach, we could confirm the flow cytometry data by demonstrating that AAV DNA was detected in all muscle-residing mononuclear cells to some extent (Figure 2E). In agreement with our flow cytometry results, the cell type with the fewest AAV DNA+ cells were *Ptprc*-expressing immune cells for all AAVs (Figure S5C). In AAV8-injected mice, FAPs showed the highest co-localization with AAV DNA (Figure S5C) and the highest proportion of tdTomato+ cells (Figure 2B, upper panel). AAVMYO- and AAVMYO2-injected mice, however, had the highest co-localization of AAV DNA with MuSCs (Figure S5C), which is different from the flow cytometry data in which endothelial cells contained the highest proportion of tdTomato+ cells (Figures 2C and 2D, upper panels).

The difference between the transgene's DNA and protein levels could have multiple origins. First and foremost, the detection of tdTomato antisense strand does not differentiate between transcribed double-stranded DNA and non-transcribed single-stranded DNA.[28] Degradation of single-stranded DNA could also lead to a loss of AAV DNA signal that contradicts the abundance of tdTomato protein. Consequently, the observation that tdTomato protein is highly abundant in AAVMYO-injected mice but its DNA is not (Figure 2E), suggests that AAVMYO may have different kinetics compared with AAV8 or AAVMYO2. In addition, transcriptional activity[29] and protein synthesis rates[30] are not accounted for in the smFISH experiments and may further contribute to discrepancies between the proportion of cells containing AAV DNA and the proportion of cells producing tdTomato protein. Overall, the smFISH experiment confirms that AAV8, AAVMYO, and AAVMYO2 can deliver the tdTomato transgene to muscle-residing mononuclear cells but does not provide quantitative measurements of muscle-residing mononuclear cell transduction. Flow cytometry, however, relies on the completion of all steps, from AAV transduction to protein synthesis, and therefore provides reliable insight into the proportion of cells that produce a protein from an AAV-delivered transgene *in vivo*.

We also analyzed whether AAV administration affected the relative proportions of the different cell types in the TA. While there were no significant differences at the low titer, we observed that the proportion of CD45+ cells was significantly higher in AAVMYO-treated mice compared with AAV8, AAVMYO2, and PBS-injected mice at the high titer of 1×10^{11} vg per TA (Figure S5D). This increase in the number of immune cells in AAVMYO-treated mice was also observed with smFISH (Figure S5E).

Level and cell type preference of tdTomato expression remains stable at 6 weeks post-AAV injection

Since muscle-residing mononuclear cells can divide and eventually lose the expression of non-integrating episomal DNA, we extended the collection of TAs injected IM with 1×10^{11} vg from 3 to 6 weeks post-administration (Figure 3A). At 6 weeks, AAVMYO remained the virus that led to the highest proportion of tdTomato+ cells (Figures 3B–3D and S7A). At both time points, AAVMYO-treated mice had significantly more tdTomato+ endothelial cells, immune

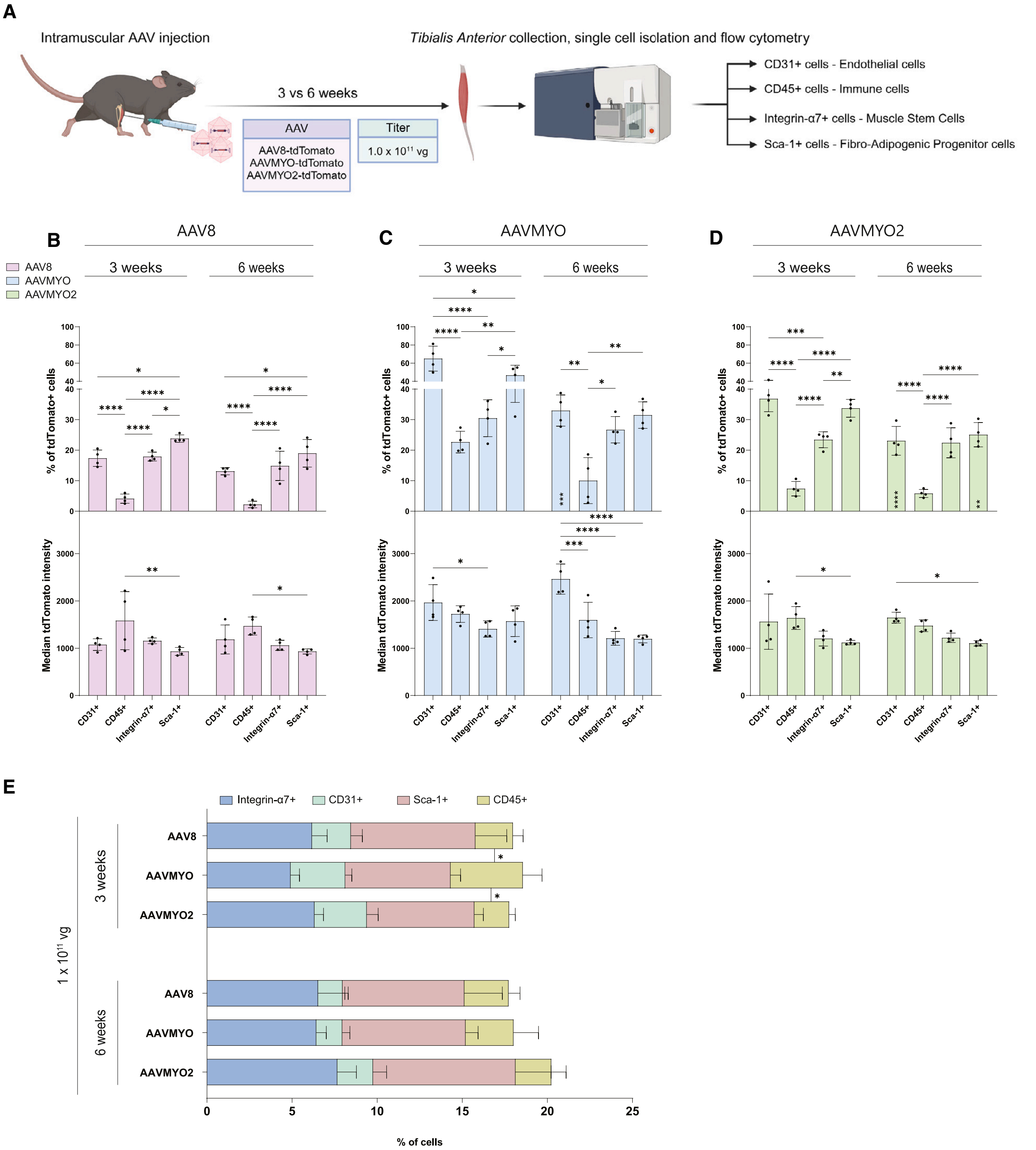

Figure 3. Quantification of AAV8, AAVMYO, and AAVMYO2 transduction levels at different time points after IM administration

(A) Schematic representation of the experimental procedure. Ten-week-old male adult C57BL/6 mice were injected IM with PBS, AAV8, AAVMYO, or AAVMYO2 at the titer of 1.0×10^{11} vg per TA (equivalent to approximately 3.3×10^{12} vg/kg) ($n = 4$ mice per condition). All viruses contained a tdTomato transgene driven by a CMV promoter to allow the labeling of transduced cells with tdTomato. Three and 6 weeks post-injection, TAs were collected and digested to generate a single-cell suspension that was stained for CD31, CD45, Integrin-α7, and Sca-1. Flow cytometry was then used to characterize the proportion of tdTomato-expressing cells and the median tdTomato signal for each mouse. (B, C, and D) AAV-specific quantifications of the proportion of tdTomato+ cells and median tdTomato signal intensity in mice treated with AAV8 (B), AAVMYO (C), and

(legend continued on next page)

cells, MuSCs, and FAPs than AAV8-treated mice (Figure S7A). The tdTomato+ endothelial cells of AAVMYO-treated mice also produced significantly more tdTomato signal than those of AAV8-treated mice at these two time points (Figure S7B). Interestingly, by delaying the tissue collection from 3 to 6 weeks post-administration, AAVMYO transduction no longer resulted in significantly higher proportions of tdTomato-expressing immune cells and FAPs compared with AAVMYO2 (Figure S7A).

Since this suggested that there were time-dependent changes in the number of tdTomato+ cells, we compared the transduction rates of each cell type at 3 and 6 weeks post-AAV administration. Overall, the proportions of tdTomato+ cells remained similar at both time points (Figures 3B–3D, upper panels). While we did not detect any significant change of the proportion of tdTomato+ cells in AAV8-injected mice (Figure 3B), AAVMYO- and AAVMYO2-injected mice showed a significant decrease in the proportion of tdTomato+ endothelial cells (Figures 3C and 3D, upper panels) (from 65.0% at 3 weeks to 33.0% at 6 weeks for AAVMYO; from 36.8% at 3 weeks to 23.1% at 6 weeks for AAVMYO2). In AAVMYO2-injected mice, there was also a significant reduction in the proportion of tdTomato+ FAPs (Figure 3D, upper panel) (from 33.7% at 3 weeks to 25.1% at 6 weeks). The median tdTomato signal emitted by transduced cells, however, was not significantly changed in any condition (Figures 3B–3D, lower panels).

Finally, the increase in the number of immune cells in AAVMYO-injected TA after 3 weeks (Figures S5D and S5E) was not present anymore 6 weeks post-administration (Figure 3E) (2.8% with AAVMYO; 2.1% with AAV8; 2.6% with AAVMYO2). This indicates that the increase in CD45+ immune cells observed 3 weeks post-AAVMYO administration is transient, not long-lasting.

Taken together, these results demonstrate that there are only a few, minor changes in mononuclear cell transduction from 3 to 6 weeks post-AAV administration. Thus, transduction of mononuclear cells in skeletal muscle seems rather stable over time. One possibility might be that cellular transduction, which may still occur between 3 and 6 weeks post-AAV administration, may at least counteract the loss of some episomes in dividing cells.

AAVMYO targets muscle-residing mononuclear cells in a dystrophic mouse model

Since the delivery of transgenes to muscle-residing mononuclear cells could provide therapeutic opportunities to treat muscular dystrophies,[21] we investigated whether AAVMYO could also transduce cells in a dystrophic mouse model with altered skeletal muscle composition. We used the dy^W/dy^W mouse model[31] of LAMA2-related muscular dystrophy (LAMA2 MD) as these mice closely recapitulate the symptoms seen in humans[32] and the cellular constitution of their muscles differs from that of wild-type mice.[12] To determine if muscle-residing mononuclear cells can be targeted in these mice, we performed IM injections of 1×10^{10} vg of AAVMYO per TA in dy^W/dy^W mice and their healthy wild-type littermates at 5 weeks of age. Three weeks later, we collected the TAs (Figure 4A) and confirmed the presence of tdTomato protein within the muscle by immunostaining (Figure 4B). Using the aforementioned smFISH approach, we detected AAV DNA in muscles from both mice (Figure 4B). Co-detection of AAV DNA and cell-specific markers revealed that endothelial cells, immune cells, MuSCs, and FAPs all co-localized with AAV DNA in wild-type and dy^W/dy^W mice (Figure 4B). Upon quantification, there were no significant differences in the transduction of the muscle-residing mononuclear cells between wild-type and dy^W/dy^W mice, although there was a trend toward higher transduction in wild-type mice (Figure 4C). In both mice, the lowest co-localization with AAV DNA was seen in the *Ptrpc*+ immune cells (13.7% in wild-type mice; 7.0% in dy^W/dy^W mice) and the highest was observed in *Pax7*+ MuSCs (30.0% in wild-type mice; 18.4% in dy^W/dy^W mice) followed closely by *Pdgfra*+ FAPs (28.8% in wild-type mice; 17.9% in dy^W/dy^W mice) (Figure 4C). All in all, these data demonstrate that AAVMYO can deliver transgenes to muscle-residing mononuclear cells in healthy and dystrophic muscle.

DISCUSSION

In this article, we show that AAV8, AAVMYO, and AAVMYO2 can transduce CD31+ endothelial cells, CD45+ immune cells, Integrin-α7+ MuSCs, and Sca-1+ FAPs in the TA. Although the efficiency of transduction varies between the serotypes, these findings have important implications for the various settings in which AAVs are employed to target skeletal muscle.

In a clinical context, our results highlight the possibility of targeting mononuclear cells with AAVs for gene therapies. MuSCs, for example, are compelling candidates for gene-editing therapies due to their successful transduction by i.v.-injected AAVs (Figures 1B–1D) and their capacity to contribute nuclei to muscle fibers. The combination of AAVs and transgene expression driven by ubiquitous promoters could increase the chances of successful genetic correction in muscle fibers by achieving editing in both fibers and MuSCs that fuse to fibers. Furthermore, in diseases such as Collagen VI-related myopathy, where the affected gene is largely expressed in FAPs,[11] gene-editing enzymes must be delivered to this cell population to restore the production of functional Collagen VI. An advantage of such gene-editing approaches is that dividing cells will generate progeny containing the edited DNA sequence. The cell division-induced loss of episomal DNA is therefore not problematic, as temporary expression of the transgene is sufficient for the therapy to exert beneficial outcomes.

AAVMYO2 (D) at 1.0×10^{11} vg per TA and collected 3 or 6 weeks post-administration. (E) Quantification of the proportion of CD31+, CD45+, Integrin-α7+, and Sca-1+ cells detected during flow cytometry. $n = 4$. Data are shown as means ± SD. Statistical significance was determined by two-way ANOVAs with Tukey's multiple comparisons test and time effects are shown by * within the bars at the 6-week time point. *$p < 0.05$, **$p < 0.01$, ***$p < 0.001$, ****$p < 0.001$. Experimental scheme (A) was created with BioRender.com.

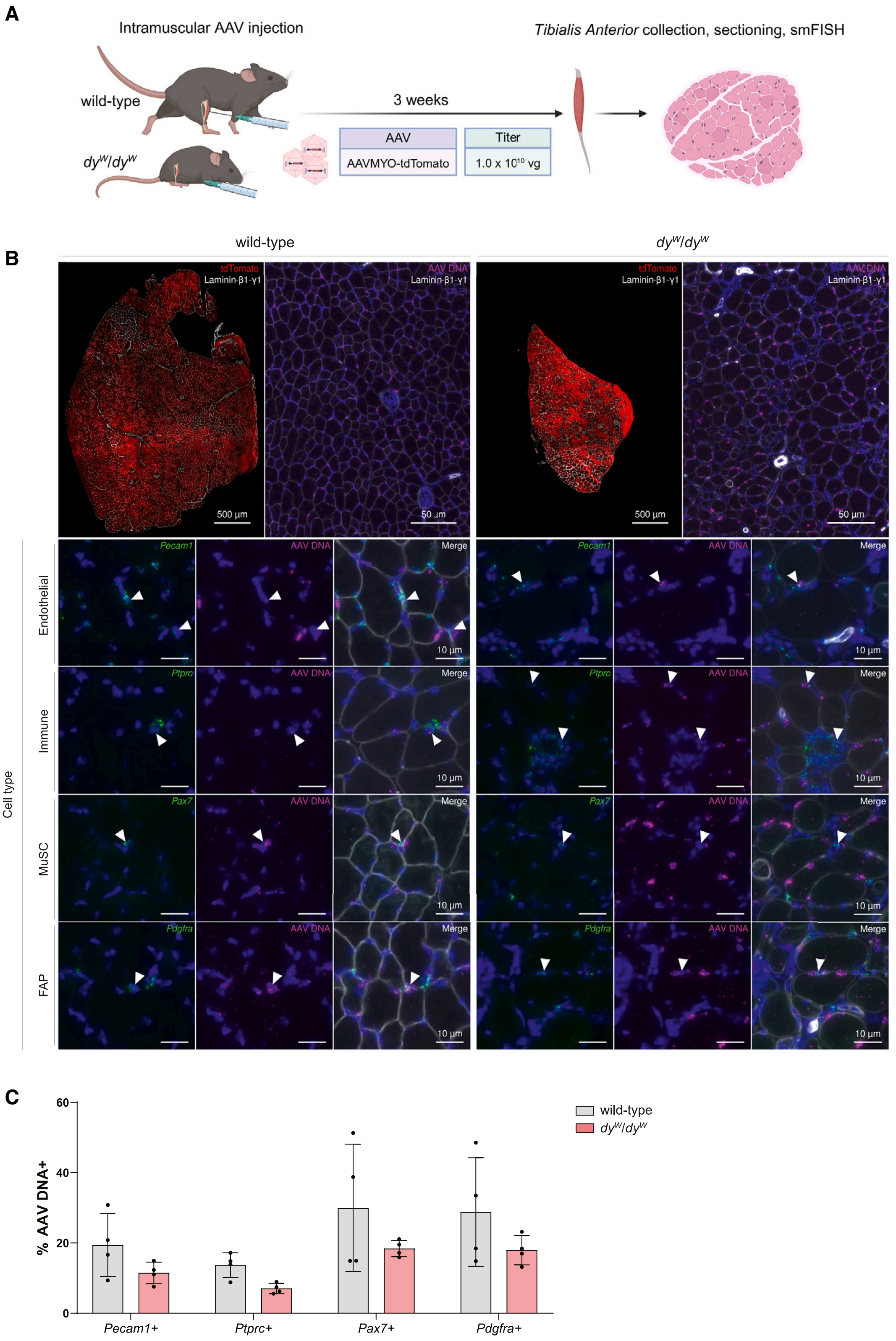

(legend on next page)

In a research context, however, the use of a ubiquitous promoter may lead scientists to draw false conclusions about a gene's function in muscle fibers. Indeed, in skeletal muscle fiber gene interrogation studies, myotropic AAVs are commonly used to overexpress or knockdown genes of interest.[2–5] We now demonstrate that the combination of AAVs and ubiquitous promoters can result in transduction of endothelial cells, immune cells, MuSCs, and FAPs. Gene modulations in these cell types may provoke muscular phenotypes of inflammation, regeneration, or fibrosis, that are wrongfully attributed to gene modulation in the fibers. This could happen, for example, when AAVs and ubiquitous promoters are employed to overexpress a gene that influences cell metabolism, as this can directly alter the state and fate of immune cells,[33] MuSCs,[34] FAPs,[35] and endothelial cells.[36] In conclusion, our data highlight the importance of combining AAVs with fiber-specific promoters when interrogating gene function in muscle fibers.

While our data demonstrated that all AAVs were capable of transducing CD31+, CD45+, Integrin-α7+, and Sca-1+ cells, the proportions of tdTomato-expressing cells varied in a cell-dependent, administration-dependent, dose-dependent, and AAV-dependent manner. In both our i.v. and IM experiments, injections of the lower titers led to very few AAV-dependent differences in the proportion of tdTomato+ cells (Figure S3A and S5A). This came as a surprise, as at low titers, AAVMYO and AAVMYO2 show higher transduction of skeletal muscle than AAV8 after i.v.[7] and IM[9] injection. We propose that these differences in tdTomato expression originate from the next-generation AAVs' increased targeting of muscle fibers, not mononuclear cells. In i.v.-injected mice, the increased targeting of muscle fibers is likely also a consequence of de-targeting of other organs, such as the liver.[7]

With the injection of the high dose, AAVMYO2 only surpassed AAV8 in the transduction rates of CD31+ cells when injected intramuscularly (Figure S5A). No other significant differences were noted between AAVMYO2 and AAV8 at the high titers (Figures S3A, S5A, and S7A). This reinforces the idea that the higher transgene expression achieved by AAVMYO2 compared with AAV8 in skeletal muscle[7] is not due to higher targeting of endothelial cells, immune cells, MuSCs, or FAPs. AAVMYO, however, outperformed AAV8 in all cell populations and resulted in a higher proportion of tdTomato+ cells (Figures S3A and S5A) and in some cases, a higher median tdTomato signal (Figures S3B and S5B). Since this did not occur at the low dose, we hypothesize that AAVMYO may saturate the muscle fibers at the high dose, enabling more transduction of mononuclear cells.[6] This could explain why AAVMYO-treated mice have higher amounts of tdTomato+ cells than AAVMYO2-treated mice (Figures S3A and S5A), as AAVMYO was demonstrated to achieve higher transduction of skeletal muscle than AAVMYO2.[7] If the greater transduction efficiency seen with AAVMYO at the high titers were due to an increased targeting toward mononuclear cells, then we would also expect to see these differences after the injection at the low dose.

Although we observed AAV-dependent differences in the number of tdTomato-expressing cells, we also observed cell-specific transduction patterns that were common to the AAVs independently of the administration route. CD45+ cells, for example, were the population with the lowest proportion of tdTomato+ cells for all AAVs at all titers (Figures 1B–1D, 2B–2D, and 3B–3D). For AAV8 and AAVMYO2, even a 10-fold increase in amount of virus injected was unable to cause a significant increase in the proportion of tdTomato-positive CD45+ cells (Figures 1B, 1D, 2B, and 2D). The only significant concentration-dependent increase in tdTomato-expressing CD45+ cells was seen in mice that received AAVMYO IM (Figure 3C). Surprisingly, the TAs from these mice also showed a dose-dependent increase in the proportion of CD45+ cells 3 weeks post-AAV administration, leading to a significant shift in their mononuclear cell composition compared with the TAs of mice that received high doses of AAV8, AAVMYO2, or PBS (Figures S5D and S5E). This increase was transient, as it was no longer observed 6 weeks post-administration (Figure 3E). By then, the proportion of tdTomato-positive CD45+ cells had fallen from 23% at 3 weeks to 10% at 6 weeks (Figure 3C). Additionally, the proportion of tdTomato-positive CD31+ cells decreased from 65% to 33% during this time (Figure 3C). This highlights that the immune response observed 3 weeks post-AAVMYO administration could have multiple origins, including the transduction of CD45+ cells, the abundance of AAVMYO capsid or a potential immunogenic effect caused by high levels of tdTomato protein (Figure 2E). In the future, administering empty AAVMYO capsids may help determine whether this transient increase in immune cells depends on the tdTomato transgene, or whether the capsid itself is sufficient to trigger this response. If that is the case, then researchers should use an empty AAVMYO capsid control rather than a PBS control for studies employing AAVMYO to modulate gene expression in skeletal muscle. Such a control would avoid wrongly attributing phenotypes of inflammation to the expression of the transgene.

Figure 4. AAVMYO can deliver transgenes to muscle-residing mononuclear cells in the dy^W/dy^W mouse model of LAMA2 MD

(A) Schematic representation of the experimental procedure. 5-week-old dy^W/dy^W mice and their wild-type littermates were injected IM with AAVMYO at the titer of 1.0×10^{10} vg per TA (equivalent to approximately 5.0×10^{11} vg/kg for wild-type mice and approximately 1.25×10^{12} vg/kg in dy^W/dy^W mice). The virus contained a tdTomato transgene driven by a CMV promoter. Three weeks post-injection, TAs were collected and smFISH was performed to detect AAV DNA and cell-specific markers. (B) Mid-belly cross-sections of wild-type and dy^W/dy^W TAs after IM injection of 1.0×10^{10} vg of AAVMYO. Upper panels, left: overview of staining for tdTomato (red) and Laminin-β1-γ1 (white). Upper panels, right: smFISH to detect *tdTomato* antisense strand (AAV DNA, magenta) and immunostaining for Laminin-β1-γ1 (white) and DAPI (blue). Note the presence of many AAV DNA-positive nuclei. Lower panels (cell type): smFISH to detect *tdTomato* antisense strand (AAV DNA) and mRNA of cell markers for endothelial cells (*Pecam1*), immune cells (*Ptprc*), MuSCs (*Pax7*), and FAPs (*Pdgfra*). DAPI (blue) was used to outline nuclei. Arrowheads indicate nuclei that are positive for the cell marker and viral DNA, demonstrating successful transduction. (C) Quantification of the proportion of cell types co-localizing with AAV DNA. $n = 4$. Data are shown as means ± SD. Statistics were evaluated using unpaired Student's t tests. Experimental scheme (A) was created with BioRender.com.

Despite CD45+ cells being the population with the lowest transduction rates for all AAVs after IM and i.v. injections, the populations with the highest transduction rates varied in an administration-dependent manner. With AAVMYO and AAVMYO2, for example, MuSCs saw significantly more tdTomato expression than endothelial cells after i.v. injection (Figures 1C and 1D), but this was reversed with IM injection (Figures 2C and 2D). We hypothesize that these differences are due to the availability of the AAVs, as IM injections are more likely to saturate the muscle fibers, thereby enabling higher transduction of mononuclear cells. Interestingly, the superior tdTomato expression achieved in endothelial cells compared with MuSCs with AAVMYO and AAVMYO2 was eliminated when tissue collection was extended from 3 to 6 weeks post-administration (Figures 3C and 3D). This was not due to an increase in the number of tdTomato-expressing MuSCs, rather a decrease in the proportion of tdTomato+ endothelial cells (Figures 3C and 3D). Possible reasons for the time-dependent decreases in the proportion of tdTomato-expressing cells could include cell apoptosis or proliferation-induced loss of episomal DNA. The latter is an important limitation of targeting muscle-residing mononuclear cells with AAVs for the sustained expression of a therapeutic transgene, particularly in muscular dystrophies with abundant tissue remodeling. This loss of episomal DNA may explain the lower number of cells co-localizing with AAV DNA in dy^W/dy^W mice, which constantly undergo cycles of degeneration and regeneration,[12,32] compared with their wild-type littermates (Figure 4C).

While quantification of tdTomato-expressing cells by flow cytometry depicts the proportion of cells producing AAV-delivered transgene product *in vivo*, it may not reflect the proportion of cells that successfully received the transgene. Certain rate-limiting steps of the AAV transduction pathway, such as AAV uncoating,[37] may differ between AAV serotypes and lead to discrepancies between the proportion of cells containing AAV DNA (Figure S3C) and the proportion of tdTomato+ cells detected by flow cytometry (Figure S5A). There are also factors that are independent of the AAV capsid that can lead to such discrepancies: some have shown, for example, that the CMV promoter has low efficiency in hematopoietic cells.[38,39] This may contribute to immune cells consistently being quantified as the population with the lowest number of tdTomato+ cells (Figures 1B–1D, 2B–2D, and 3B–3D). In agreement with this hypothesis, there were more immune cells co-localizing with AAV DNA in our smFISH experiment (Figure S5C) than tdTomato+ immune cells in our flow cytometry experiment (Figure S5A). However, this pattern was not specific to immune cells (Figures S5A and S5C) and immune cells were consistently the population with the fewest cells containing AAV DNA (Figure S5C) and protein (Figure S5A). Furthermore, as we discussed above, the presence of AAV DNA does not imply that the transgene is expressed, as viral DNA can be present in infected cells in a latent state or as a concatemer,[40] indicating that smFISH cannot conclusively determine whether the use of a CMV promoter is hindering tdTomato transcription in immune cells. Additionally, there is a possibility that we overestimated the number of transduced immune cells due to nonspecific uptake of tdTomato protein. Indeed, phagocytosis of transduced muscle fibers, FAPs, MuSCs, and endothelial cells could result in non-transduced immune cells being detected as tdTomato+.[41] It seems, however, that this uptake is likely a minor contributor to the expression of tdTomato as the smFISH data indicate that expression of tdTomato in individual cell types correlates well with the presence of viral DNA.

In future studies, the quantifications of tdTomato+ cells should be repeated using mice from different backgrounds. Here, we only quantified the proportions of tdTomato-expressing cells by flow cytometry in male adult C57BL/6 mice. As it has been demonstrated that skeletal muscle transduction rates vary between genetic backgrounds,[42] one may also observe background-dependent variations in mononuclear cell transduction. It is also important to highlight that we only quantified transduction rates in cells from the TA to ensure that data were comparable between i.v. and IM injections. It is possible that cells from different muscles have different transduction rates. Finally, for the quantification of tdTomato+ cells by flow cytometry, we focused on cells that were only positive for one cell type marker. It is possible that certain cells stained positive for multiple markers and were omitted from the analyses. Altogether, our data provide insight into the specificities and effectiveness of different AAV capsids in transducing muscle-residing mononuclear cells after IM and i.v. administration. These results are an important guidance for researchers developing gene therapies or interrogating gene function in skeletal muscle via AAVs.

MATERIALS AND METHODS

Ethical statement

All procedures involving animals were performed in accordance with Swiss regulations and approved by the veterinary commission of the canton Basel-Stadt.

Mice

C57BL/6JRj mice were ordered from Janvier Labs (Saint-Berthevin, France) and kept on a 12-h light-dark cycle (6 a.m.–6 p.m.) at 22°C (range 20–24°C) and 55% (range 45%–65%) relative humidity. All C57BL/6JRj mice used in the experiments were male and between 8 and 10 weeks of age at the time of the AAV administration.

To test AAV transduction in a dystrophic mouse model, we administered AAVs to the dy^W/dy^W mouse model of LAMA2 MD[43] (B6.129S1(Cg)-Lama2<tm1Eeng>; Jackson Laboratory stock #013786) and their wild-type healthy littermates at 5 weeks of age. Long-necked water bottles and wet food were given to all cages to ensure dystrophic mice could access food and water.

AAV production, purification, and titration

All AAV transfer vectors contained a ubiquitous CMV promoter, tdTomato, WPRE ,and bovine growth hormone polyA signal. Plasmids are available upon demand.

AAVs were produced in-house. For this, HEK293T cells (CRL-3216, ATCC, Manassas, Virginia) were transfected with transfer plasmid

(AAV-tdTomato construct), AAV helper (AAV8[7]/AVMYO[6]/AAV-MYO2[7]) and pAdDeltaF6 helper plasmid (a gift from J. M. Wilson Addgene, plasmid # 112867) using PEI MAX (Polyscience, Warrington, Pennsylvania). Forty 15-cm tissue culture plates were processed for each preparation. Briefly, the supernatant was collected at 48 and 72 h post-transfection. Cells were then dislodged at 72 h post-transfection with PBS, before being centrifuged at 500 × *g* at 4°C for 10 min and resuspended in AAV lysis solution (50 mM Tris-HCl, 1 M NaCl, 10 mM $MgCl_2$, pH 8.5). A total of 1,000 U of salt active nuclease (Sigma, Burlington, Massachusetts) was added to the cells for an incubation at 37°C for 1 h. The samples were vortexed every 10 min. Samples were then centrifuged at 4,000 × *g* for 15 min at 4°C. The supernatant was collected for AAV precipitation. Polyethylene glycol 8000 (Sigma, Burlington, Massachusetts) was added to the HEK293T cells' supernatant to a final concentration of 8% (w/v). Samples were incubated for 2 h at 4°C before centrifugation at 4,000 × *g* for 30 min at 4°C. The pellet was then resuspended in AAV lysis buffer and pooled with the cell lysate. A 15%–25%–40%–60% iodixanol (Serumwerk, Bernburg, Germany) gradient was then used to purify AAV particles. The gradient was centrifuged at 292,000 × *g* (Beckman type 70 Ti rotor) for 2 h at 4°C and the AAV particles were collected from the 40%–60% phase interface. The extract was filtered through a 100 kDa MWCO filter (Millipore, Burlington, Massachusetts) and washed with PBS containing 0.01% Pluronic F-68 surfactant (Gibco, Carlsbad, California) until the buffer exchange was complete. Final volume was decreased to reach a final AAV concentration >1×10^{13} vg/mL. AAVs were titrated at the same time via digital PCR with the ITR2/5 assay (QIAGEN, Hilden, Germany).

AAV administration

For intramuscular injections, mice were anesthetized with isoflurane and injected with 30 μL of AAV (1.0×10^{10} or 1.0×10^{11} vg) or PBS in both TAs. For intravenous injections, 100 μL of AAV (3.0×10^{12} or 3.0×10^{13} vg/kg) or PBS was injected into the lateral tail vein. Mice were euthanized at 3 or 6 weeks post-injection.

Flow cytometry and fluorescence-activated cell sorting

TA muscles were collected in 500 μL of Ham's F-10 media (Gibco, Carlsbad, California) and minced using scissors. Minced TAs were then transferred to a GentleMACS C tube (Miltenyi, Bergisch Gladbach, Germany) and 5 mL of pre-warmed digestion buffer (1% Collagenase B (Roche, Basel, Switzerland), 0.4% Dispase II (Roche, Basel, Switzerland), and 25 mM HEPES (Bioconcept, Allschwil, Switzerland) in Ham's F-10 media (Gibco, Carlsbad, California) was added. Tubes were then mounted on a GentleMACS Octo Separator (Miltenyi, Bergisch Gladbach, Germany) and the following program was run: Temp ON 37°C, 90 rpm 5 min, −90 rpm 5 min, loop 10x (360 rpm 5 s, −360 rpm 5 s), end loop, 90 rpm 5 min, −90 rpm 5 min, end. Five milliliters of FBS (Biological Industries, Kibbutz Beit Haemek, Israel) was then added to halt the digestion and the content of the tube was transferred to a 50-mL conical tube through a 70-μm filter that was subsequently washed using 20 mL of cold flow cytometry buffer (2% FBS, 2.5 mM EDTA in PBS). The samples were then centrifuged for 5 min at 500 × *g* and 4°C before being resuspended in 500 μL of cold flow cytometry buffer. Two milliliters of red blood cell lysing buffer (Sigma, Burlington, Massachusetts) was added for 5 min at room temperature (RT), the lysates were then diluted in 10 mL of cold flow cytometry buffer and centrifuged for 5 min at 500 × *g* and 4°C. The cells were then resuspended in flow cytometry buffer and stained for 10 min at 4°C (see Table S7). After the staining, 10 mL of cold flow cytometry buffer was added to the cells and the tubes were centrifuged at 500 × *g* and 4°C for 5 min. Finally, the cells were resuspended in cold flow cytometry buffer, strained through a 40-μm filter, and analyzed using a FACSAria Fusion (BD Biosciences, Franklin Lakes, New Jersey). Gates were set from analyses of single stains and fluorescence-minus-one stains (Figure S1). Flow cytometry data were analyzed using FlowJo v10.8 Software (BD Life Sciences, Franklin Lakes, New Jersey).

Immunostaining

For immunostaining, TAs were collected and placed in 4% PFA for 2 h at 4°C. They were then transferred to 20% sucrose and left overnight at 4°C before embedding in optimal cutting temperature (Tissue-Tek O.C.T., Torrance, California) and freezing in cooled isopentane for 20 s. Ten-micrometer sections were cut from the middle of the TA at −20°C on a cryostat (Leica, CM1950, Wetzlar, Germany) and collected on SuperFrost Plus (VWR, Radnor, Pennsylvania) slides. The sections were fixed in 4% PFA for 5 min at RT, washed in PBS, and blocked in 10% goat serum (Biological Industries, Kibbutz Beit Haemek, Israel) in 0.1% Tween 20 in PBS. They were then incubated in primary antibody solution (see Table S7) overnight at 4°C, washed four times for 5 min in PBS, incubated in a secondary antibody solution (see Table S7) for 1 h at RT, washed four times for 5 min in PBS and mounted using ProLong Gold antifade with DAPI (Invitrogen, Waltham, Massachusetts).

Single molecule fluorescence *in situ* hybridization (smFISH; RNAscope)

For smFISH, TAs were collected and immediately embedded in optimal cutting temperature (Tissue-Tek O.C.T., Torrance, California) and frozen in cooled isopentane for 20 s before storage at −80°C. Ten-micrometer sections were cut from the middle of the TAs at −20°C on a cryostat (Leica, CM1950, Wetzlar, Germany) and collected on SuperFrost Plus (VWR, Radnor, Pennsylvania) slides. Sections were fixed in 4% PFA for 1 h at 4°C, washed two times for 1 min in PBS before serial dehydration for 5 min in 50% ethanol, 70% ethanol, and twice in 100% ethanol. The slides were stored overnight at −20°C in 100% ethanol. The next day, slides were dried for 3 min at RT and sections were circled with a Hydrophobic Barrier Pen (Vector Laboratories, Newark, California) before 30 min of protease digestion (Protease IV) at RT. Slides were then washed three times for 2 min in ddH_2O. Hybridization (probe catalog numbers in Table S8) and subsequent amplification steps were performed according to the manufacturer's instructions for the Multiplex V2 assay (323100, Advanced Cell Diagnostics, Newark, California) at 40°C in a HybEZ oven (Advanced Cell Diagnostics, Newark, California). TSA vivid 520 (323271), 570 (323272), and 650 (323273) fluorophores

were purchased from Advanced Cell Diagnostics (Newark, California). After hybridization, slides were blocked for 30 min at RT in 0.4% Triton X-100 and 3% bovine serum albumin in PBS. They were then incubated overnight at 4°C with Laminin-β1-γ1 primary antibody (see Table S7), washed four times in PBS for 5 min and incubated in secondary antibody (see Table S7) for 1 h at RT. Finally, slides were washed four times for 5 min in PBS and mounted with ProLong Gold antifade with DAPI (Invitrogen, Waltham, Massachusetts). Full TA cross-sections were imaged on a Zeiss Axio Scan.Z1 (Zeiss, Oberkochen, Germany).

Statistical analyses

All statistical methods used to analyze the data are described in the figure legends. One-way analysis of variance (ANOVA), two-way ANOVAs mixed effect models with repeated measures followed by Tukey's *post hoc* test, and unpaired Student's t tests were performed using GraphPad Prism 9 software. When quantifying tdTomato signal with missing values (no transduction), a main-effect-only model was fit. Statistical significance was set at a p value <0.05. Error bars represent standard deviation, as indicated in legends.

DATA AVAILABILITY

The flow cytometry data that support this study can be found in Tables S1–S6. More information is available on request from the corresponding author (M.A.R.).

ACKNOWLEDGMENTS

We would like to thank the Biozentrum FACS Core facility and Animal Housing facility for their technical support. We would also like to thank Prof. Dirk Grimm for providing the plasmids for AAVMYO and AAVMYO2 capsids. T.J.M. was supported by a Biozentrum PhD Fellowship for Excellence and the remaining support awarded to M.A.R. originated from grants from the European Joint Programme on Rare Diseases (MYOCITY), the Swiss National Science Foundation (grant number 169845), and from the cantons of Basel-Stadt and Basel-Landschaft.

AUTHOR CONTRIBUTIONS

Conceptualization: T.J.M. and M.A.R.; methodology: T.J.M.; investigations: T.J.M., N.L., L.J., E.M., and M.T.; statistical analysis: T.J.M.; visualization: T.J.M.; supervision: M.A.R.; writing – original draft: T.J.M. and M.A.R.; writing – review and editing: T.J.M. and M.A.R.; funding acquisition: M.A.R.

DECLARATION OF INTERESTS

M.A.R is the founder and CEO of SEAL Therapeutics Ltd.

SUPPLEMENTAL INFORMATION

Supplemental information can be found online at https://doi.org/10.1016/j.omtm.2025.101451.

REFERENCES

1. Skopenkova, V.V., Egorova, T.V., and Bardina, M.V. (2021). Muscle-Specific Promoters for Gene Therapy. Acta Naturae *13*, 47–58. https://doi.org/10.32607/acta-naturae.11063.
2. Brearley-Sholto, M.C., Loczenski-Brown, D.M., Jones, S., Daniel, Z.C.T.R., Ebling, F.J.P., Parr, T., and Brameld, J.M. (2021). Effect of AAV-mediated overexpression of ATF5 and downstream targets of an integrated stress response in murine skeletal muscle. Sci. Rep. *11*, 19796. https://doi.org/10.1038/s41598-021-99432-4.
3. Loczenski-Brown, D.M., Jones, S., Luckett, J., Daniel, Z., Brearley, M.C., Ebling, F.J.P., Parr, T., and Brameld, J.M. (2019). Effect of adeno-associated virus (AAV)-mediated overexpression of PEPCK-M (Pck2) on Clenbuterol-induced muscle growth. PLoS One *14*, e0218970. https://doi.org/10.1371/journal.pone.0218970.
4. Tasfaout, H., Lionello, V.M., Kretz, C., Koebel, P., Messaddeq, N., Bitz, D., Laporte, J., and Cowling, B.S. (2018). Single Intramuscular Injection of AAV-shRNA Reduces DNM2 and Prevents Myotubular Myopathy in Mice. Mol. Ther. *26*, 1082–1092. https://doi.org/10.1016/j.ymthe.2018.02.008.
5. Guo, M., Zhang, J., Ma, Y., Zhu, Z., Zuo, H., Yao, J., Wu, X., Wang, D., Yu, J., Meng, M., et al. (2023). AAV-Mediated nuclear localized PGC1alpha4 delivery in muscle ameliorates sarcopenia and aging-associated metabolic dysfunctions. Aging Cell *22*, e13961. https://doi.org/10.1111/acel.13961.
6. Weinmann, J., Weis, S., Sippel, J., Tulalamba, W., Remes, A., El Andari, J., Herrmann, A.K., Pham, Q.H., Borowski, C., Hille, S., et al. (2020). Identification of a myotropic AAV by massively parallel in vivo evaluation of barcoded capsid variants. Nat. Commun. *11*, 5432. https://doi.org/10.1038/s41467-020-19230-w.
7. El Andari, J., Renaud-Gabardos, E., Tulalamba, W., Weinmann, J., Mangin, L., Pham, Q.H., Hille, S., Bennett, A., Attebi, E., Bourges, E., et al. (2022). Semirational bioengineering of AAV vectors with increased potency and specificity for systemic gene therapy of muscle disorders. Sci. Adv. *8*, eabn4704. https://doi.org/10.1126/sciadv.abn4704.
8. Tabebordbar, M., Lagerborg, K.A., Stanton, A., King, E.M., Ye, S., Tellez, L., Krunnfusz, A., Tavakoli, S., Widrick, J.J., Messemer, K.A., et al. (2021). Directed evolution of a family of AAV capsid variants enabling potent muscle-directed gene delivery across species. Cell *184*, 4919–4938.e22. https://doi.org/10.1016/j.cell.2021.08.028.
9. Thurkauf, M., Lin, S., Oliveri, F., Grimm, D., Platt, R.J., and Ruegg, M.A. (2023). Fast, multiplexable and efficient somatic gene deletions in adult mouse skeletal muscle fibers using AAV-CRISPR/Cas9. Nat. Commun. *14*, 6116. https://doi.org/10.1038/s41467-023-41769-7.
10. Schmalbruch, H., and Hellhammer, U. (1977). The number of nuclei in adult rat muscles with special reference to satellite cells. Anat. Rec. *189*, 169–175. https://doi.org/10.1002/ar.1091890204.
11. Petrany, M.J., Swoboda, C.O., Sun, C., Chetal, K., Chen, X., Weirauch, M.T., Salomonis, N., and Millay, D.P. (2020). Single-nucleus RNA-seq identifies transcriptional heterogeneity in multinucleated skeletal myofibers. Nat. Commun. *11*, 6374. https://doi.org/10.1038/s41467-020-20063-w.
12. Le Moal, E., Liu, Y., Collerette-Tremblay, J., Dumontier, S., Fabre, P., Molina, T., Dort, J., Orfi, Z., Denault, N., Boutin, J., et al. (2024). Apelin stimulation of the vascular skeletal muscle stem cell niche enhances endogenous repair in dystrophic mice. Sci. Transl. Med. *16*, eabn8529. https://doi.org/10.1126/scitranslmed.abn8529.
13. Lukjanenko, L., Karaz, S., Stuelsatz, P., Gurriaran-Rodriguez, U., Michaud, J., Dammone, G., Sizzano, F., Mashinchian, O., Ancel, S., Migliavacca, E., et al. (2019). Aging Disrupts Muscle Stem Cell Function by Impairing Matricellular WISP1 Secretion from Fibro-Adipogenic Progenitors. Cell Stem Cell *24*, 433–446.e7. https://doi.org/10.1016/j.stem.2018.12.014.
14. Mueller, A.A., van Velthoven, C.T., Fukumoto, K.D., Cheung, T.H., and Rando, T.A. (2016). Intronic polyadenylation of PDGFRalpha in resident stem cells attenuates muscle fibrosis. Nature *540*, 276–279. https://doi.org/10.1038/nature20160.
15. Feichtinger, R.G., Mucha, B.E., Hengel, H., Orfi, Z., Makowski, C., Dort, J., D'Anjou, G., Nguyen, T.T.M., Buchert, R., Juenger, H., et al. (2019). Biallelic variants in the transcription factor PAX7 are a new genetic cause of myopathy. Genet. Med. *21*, 2521–2531. https://doi.org/10.1038/s41436-019-0532-z.
16. Noviello, C., Kobon, K., Delivry, L., Guilbert, T., Britto, F., Julienne, F., Maire, P., Randrianarison-Huetz, V., and Sotiropoulos, A. (2022). RhoA within myofibers controls satellite cell microenvironment to allow hypertrophic growth. iScience *25*, 103616. https://doi.org/10.1016/j.isci.2021.103616.
17. Dumont, N.A., Wang, Y.X., von Maltzahn, J., Pasut, A., Bentzinger, C.F., Brun, C.E., and Rudnicki, M.A. (2015). Dystrophin expression in muscle stem cells regulates their polarity and asymmetric division. Nat. Med. *21*, 1455–1463. https://doi.org/10.1038/nm.3990.
18. Conte, T.C., Duran-Bishop, G., Orfi, Z., Mokhtari, I., Deprez, A., Côté, I., Molina, T., Kim, T.Y., Tellier, L., Roussel, M.P., et al. (2023). Clearance of defective muscle stem cells by senolytics restores myogenesis in myotonic dystrophy type 1. Nat. Commun. *14*, 4033. https://doi.org/10.1038/s41467-023-39663-3.

19. Kuang, W., Xu, H., Vilquin, J.T., and Engvall, E. (1999). Activation of the lama2 gene in muscle regeneration: abortive regeneration in laminin alpha2-deficiency. Lab. Invest. *79*, 1601–1613.

20. Arrighi, N., Moratal, C., Savary, G., Fassy, J., Nottet, N., Pons, N., Clément, N., Fellah, S., Larrue, R., Magnone, V., et al. (2021). The FibromiR miR-214-3p Is Upregulated in Duchenne Muscular Dystrophy and Promotes Differentiation of Human Fibro-Adipogenic Muscle Progenitors. Cells *10*, 1832. https://doi.org/10.3390/cells10071832.

21. Tabebordbar, M., Zhu, K., Cheng, J.K.W., Chew, W.L., Widrick, J.J., Yan, W.X., Maesner, C., Wu, E.Y., Xiao, R., Ran, F.A., et al. (2016). In vivo gene editing in dystrophic mouse muscle and muscle stem cells. Science *351*, 407–411. https://doi.org/10.1126/science.aad5177.

22. Sacco, A., Doyonnas, R., Kraft, P., Vitorovic, S., and Blau, H.M. (2008). Self-renewal and expansion of single transplanted muscle stem cells. Nature *456*, 502–506. https://doi.org/10.1038/nature07384.

23. Liu, L., Cheung, T.H., Charville, G.W., Hurgo, B.M.C., Leavitt, T., Shih, J., Brunet, A., and Rando, T.A. (2013). Chromatin Modifications as Determinants of Muscle Stem Cell Quiescence and Chronological Aging. Cell Rep. *4*, 189–204. https://doi.org/10.1016/j.celrep.2013.05.043.

24. Tierney, M.T., Aydogdu, T., Sala, D., Malecova, B., Gatto, S., Puri, P.L., Latella, L., and Sacco, A. (2014). STAT3 signaling controls satellite cell expansion and skeletal muscle repair. Nat. Med. *20*, 1182–1186. https://doi.org/10.1038/nm.3656.

25. Liu, L., Cheung, T.H., Charville, G.W., and Rando, T.A. (2015). Isolation of skeletal muscle stem cells by fluorescence-activated cell sorting. Nat. Protoc. *10*, 1612–1624. https://doi.org/10.1038/nprot.2015.110.

26. Elizalde, G., Munoz, A.Z., and Almada, A.E. (2023). Protocol for the isolation of mouse muscle stem cells using fluorescence-activated cell sorting. STAR Protoc. *4*, 102656. https://doi.org/10.1016/j.xpro.2023.102656.

27. Sihn, C.R., Handyside, B., Liu, S., Zhang, L., Murphy, R., Yates, B., Xie, L., Torres, R., Russell, C.B., O'Neill, C.A., et al. (2022). Molecular analysis of AAV5-hFVIII-SQ vector-genome-processing kinetics in transduced mouse and nonhuman primate livers. Mol. Ther. Methods Clin. Dev. *24*, 142–153. https://doi.org/10.1016/j.omtm.2021.12.004.

28. Zhao, J., Yue, Y., Patel, A., Wasala, L., Karp, J.F., Zhang, K., Duan, D., and Lai, Y. (2020). High-Resolution Histological Landscape of AAV DNA Distribution in Cellular Compartments and Tissues following Local and Systemic Injection. Mol. Ther. Methods Clin. Dev. *18*, 856–868. https://doi.org/10.1016/j.omtm.2020.08.006.

29. van Velthoven, C.T.J., de Morree, A., Egner, I.M., Brett, J.O., and Rando, T.A. (2017). Transcriptional Profiling of Quiescent Muscle Stem Cells In Vivo. Cell Rep. *21*, 1994–2004. https://doi.org/10.1016/j.celrep.2017.10.037.

30. Zismanov, V., Chichkov, V., Colangelo, V., Jamet, S., Wang, S., Syme, A., Koromilas, A.E., and Crist, C. (2016). Phosphorylation of eIF2alpha Is a Translational Control Mechanism Regulating Muscle Stem Cell Quiescence and Self-Renewal. Cell Stem Cell *18*, 79–90. https://doi.org/10.1016/j.stem.2015.09.020.

31. Kuang, W., Xu, H., Vachon, P.H., Liu, L., Loechel, F., Wewer, U.M., and Engvall, E. (1998). Merosin-deficient congenital muscular dystrophy. Partial genetic correction in two mouse models. J. Clin. Investig. *102*, 844–852. https://doi.org/10.1172/JCI3705.

32. Reinhard, J.R., Lin, S., McKee, K.K., Meinen, S., Crosson, S.C., Sury, M., Hobbs, S., Maier, G., Yurchenco, P.D., and Rüegg, M.A. (2017). Linker proteins restore basement membrane and correct LAMA2-related muscular dystrophy in mice. Sci. Transl. Med. *9*, eaal4649. https://doi.org/10.1126/scitranslmed.aal4649.

33. Chi, H. (2022). Immunometabolism at the intersection of metabolic signaling, cell fate, and systems immunology. Cell. Mol. Immunol. *19*, 299–302. https://doi.org/10.1038/s41423-022-00840-x.

34. Relaix, F., Bencze, M., Borok, M.J., Der Vartanian, A., Gattazzo, F., Mademtzoglou, D., Perez-Diaz, S., Prola, A., Reyes-Fernandez, P.C., Rotini, A., and Taglietti, T. (2021). Perspectives on skeletal muscle stem cells. Nat. Commun. *12*, 692. https://doi.org/10.1038/s41467-020-20760-6.

35. Reggio, A., Rosina, M., Krahmer, N., Palma, A., Petrilli, L.L., Maiolatesi, G., Massacci, G., Salvatori, I., Valle, C., Testa, S., et al. (2020). Metabolic reprogramming of fibro/adipogenic progenitors facilitates muscle regeneration. Life Sci. Alliance *3*, e202000646. https://doi.org/10.26508/lsa.202000660.

36. Li, X., Sun, X., and Carmeliet, P. (2019). Hallmarks of Endothelial Cell Metabolism in Health and Disease. Cell Metab. *30*, 414–433. https://doi.org/10.1016/j.cmet.2019.08.011.

37. Hauck, B., Zhao, W., High, K., and Xiao, W. (2004). Intracellular viral processing, not single-stranded DNA accumulation, is crucial for recombinant adeno-associated virus transduction. J. Virol. *78*, 13678–13686. https://doi.org/10.1128/JVI.78.24.13678-13686.2004.

38. Byun, H.M., Suh, D., Jeong, Y., Wee, H.S., Kim, J.M., Kim, W.K., Ko, J.J., Kim, J.S., Lee, Y.B., and Oh, Y.K. (2005). Plasmid vectors harboring cellular promoters can induce prolonged gene expression in hematopoietic and mesenchymal progenitor cells. Biochem. Biophys. Res. Commun. *332*, 518–523. https://doi.org/10.1016/j.bbrc.2005.04.155.

39. Yam, P.Y., Li, S., Wu, J., Hu, J., Zaia, J.A., and Yee, J.K. (2002). Design of HIV vectors for efficient gene delivery into human hematopoietic cells. Mol. Ther. *5*, 479–484. https://doi.org/10.1006/mthe.2002.0558.

40. Vincent-Lacaze, N., Snyder, R.O., Gluzman, R., Bohl, D., Lagarde, C., and Danos, O. (1999). Structure of adeno-associated virus vector DNA following transduction of the skeletal muscle. J. Virol. *73*, 1949–1955. https://doi.org/10.1128/JVI.73.3.1949-1955.1999.

41. Summan, M., Warren, G.L., Mercer, R.R., Chapman, R., Hulderman, T., Van Rooijen, N., and Simeonova, P.P. (2006). Macrophages and skeletal muscle regeneration: a clodronate-containing liposome depletion study. Am. J. Physiol. Regul. Integr. Comp. Physiol. *290*, R1488–R1495. https://doi.org/10.1152/ajpregu.00465.2005.

42. Ji, J., Lefebvre, E., and Laporte, J. (2024). Comparative in vivo characterization of newly discovered myotropic adeno-associated vectors. Skelet. Muscle *14*, 9. https://doi.org/10.1186/s13395-024-00341-7.

43. Kuang, W., Xu, H., Vachon, P.H., and Engvall, E. (1998). Disruption of the lama2 gene in embryonic stem cells: laminin alpha 2 is necessary for sustenance of mature muscle cells. Exp. Cell Res. *241*, 117–125. https://doi.org/10.1006/excr.1998.4025.

OMTM, Volume *33*

Supplemental information

AAV capsids target muscle-resident cells with different efficiencies—A comparative study between AAV8, AAVMYO, and AAVMYO2

Timothy J. McGowan, Nicolas Lewerenz, Eleonora Maino, Marco Thürkauf, Lena Jörin, and Markus A. Rüegg

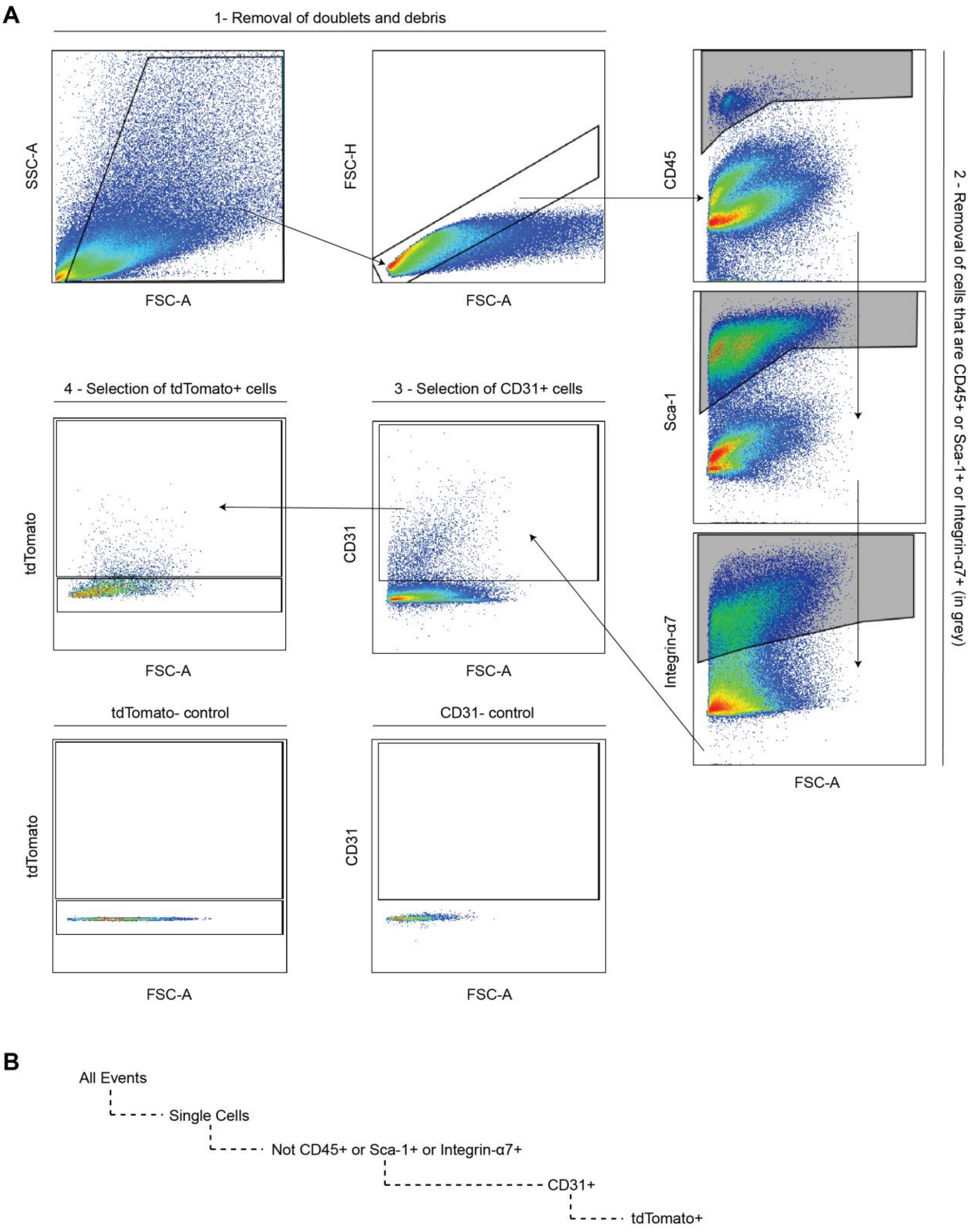
A
1- Removal of doublets and debris
SSC-A
FSC-A
FSC-H
FSC-A
CD45
Sca-1
Integrin-α7
FSC-A
2 - Removal of cells that are CD45+ or Sca-1+ or Integrin-α7+ (in grey)
4 - Selection of tdTomato+ cells
3 - Selection of CD31+ cells
tdTomato
FSC-A
CD31
FSC-A
tdTomato- control
CD31- control
tdTomato
FSC-A
CD31
FSC-A
B
All Events
Single Cells
Not CD45+ or Sca-1+ or Integrin-α7+
CD31+
tdTomato+

<u>Fig. S1: Design of the flow cytometry panel.</u> For visualization purposes, we show the flow cytometry panel design for one cell population (CD31+ cells). The following steps were applied for all cell populations. **A** We first removed cell duplicates and focused on single cells (1). Then, we removed cells that stained positive for CD45, Sca-1 or Integrin-α7 (2) and kept cells that were positive for CD31 (3). Finally, within this population, we quantified the proportion of cells that were tdTomato+ (4). A fluorescence-minus-one control in which CD31 antibody was omitted and a tdTomato-negative sample were used to set the boundaries between cells that were positive or negative for a marker. These controls are displayed under the panels showing the selection of CD31+ cells (3) and tdTomato+ cells (4), respectively. **B** Text description of the sorting strategy.

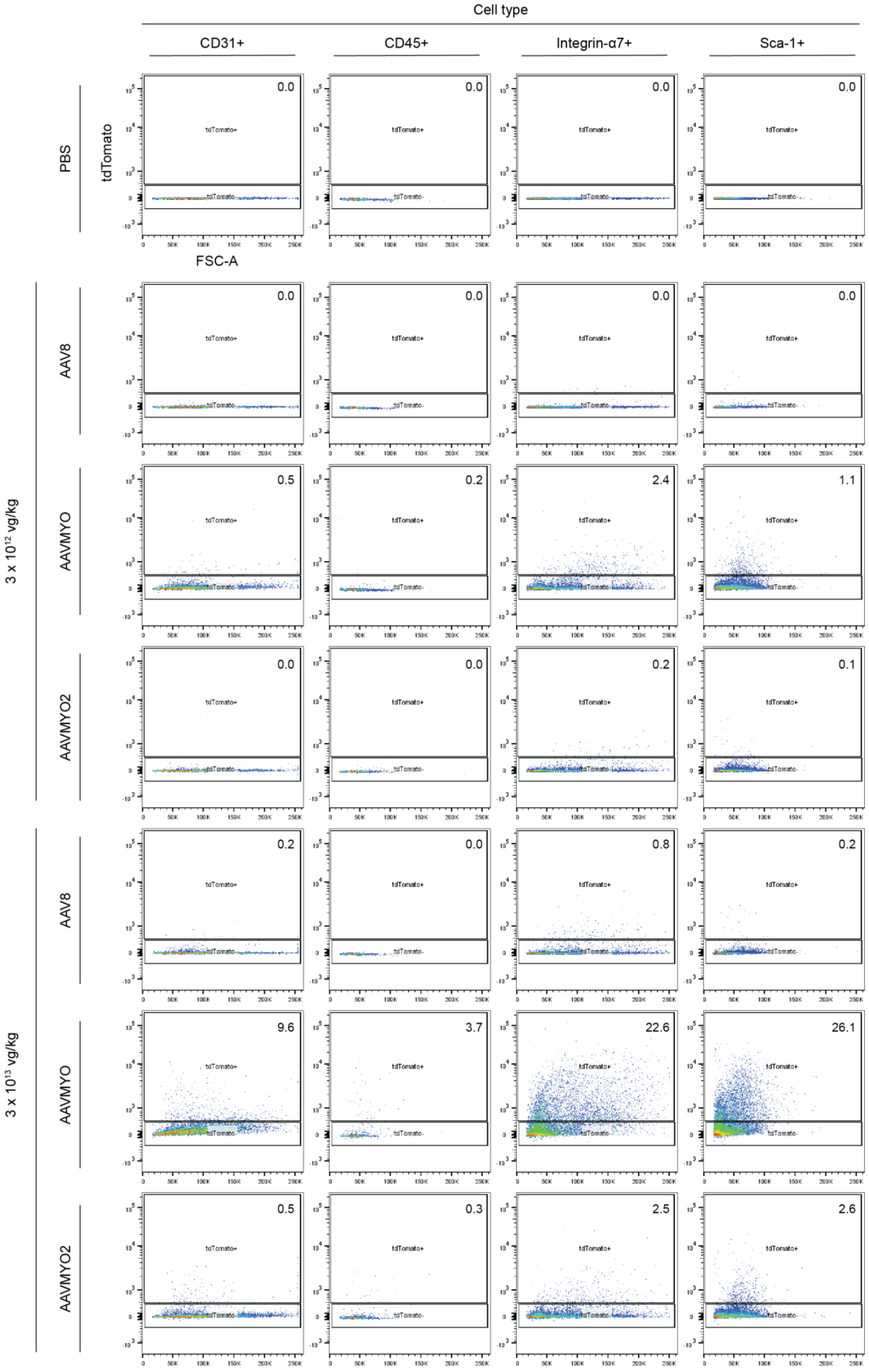

Cell type
CD31+
CD45+
Integrin-α7+
Sca-1+
PBS
tdTomato
FSC-A
tdTomato+
tdTomato-
3 x 10¹² vg/kg
3 x 10¹³ vg/kg
AAV8
AAVMYO
AAVMYO2
0.0 0.0 0.0 0.0
0.0 0.0 0.0 0.0
0.5 0.2 2.4 1.1
0.0 0.0 0.2 0.1
0.2 0.0 0.8 0.2
9.6 3.7 22.6 26.1
0.5 0.3 2.5 2.6

Fig. S2: Flow cytometry panel after IV injection of AAV8, AAVMYO and AAVMYO2. Representative panels showing tdTomato signal in CD31+, CD45+, Integrin-α7+ and Sca-1+ cells (the staining strategy to isolate these cells is shown in Fig. S1) of mice injected IV with PBS, AAV8, AAVMYO or AAVMYO2 with 3.0×10^{12} or 3.0×10^{13} vg/kg. The average proportion of tdTomato+ cells is displayed in the top right corner of each panel.

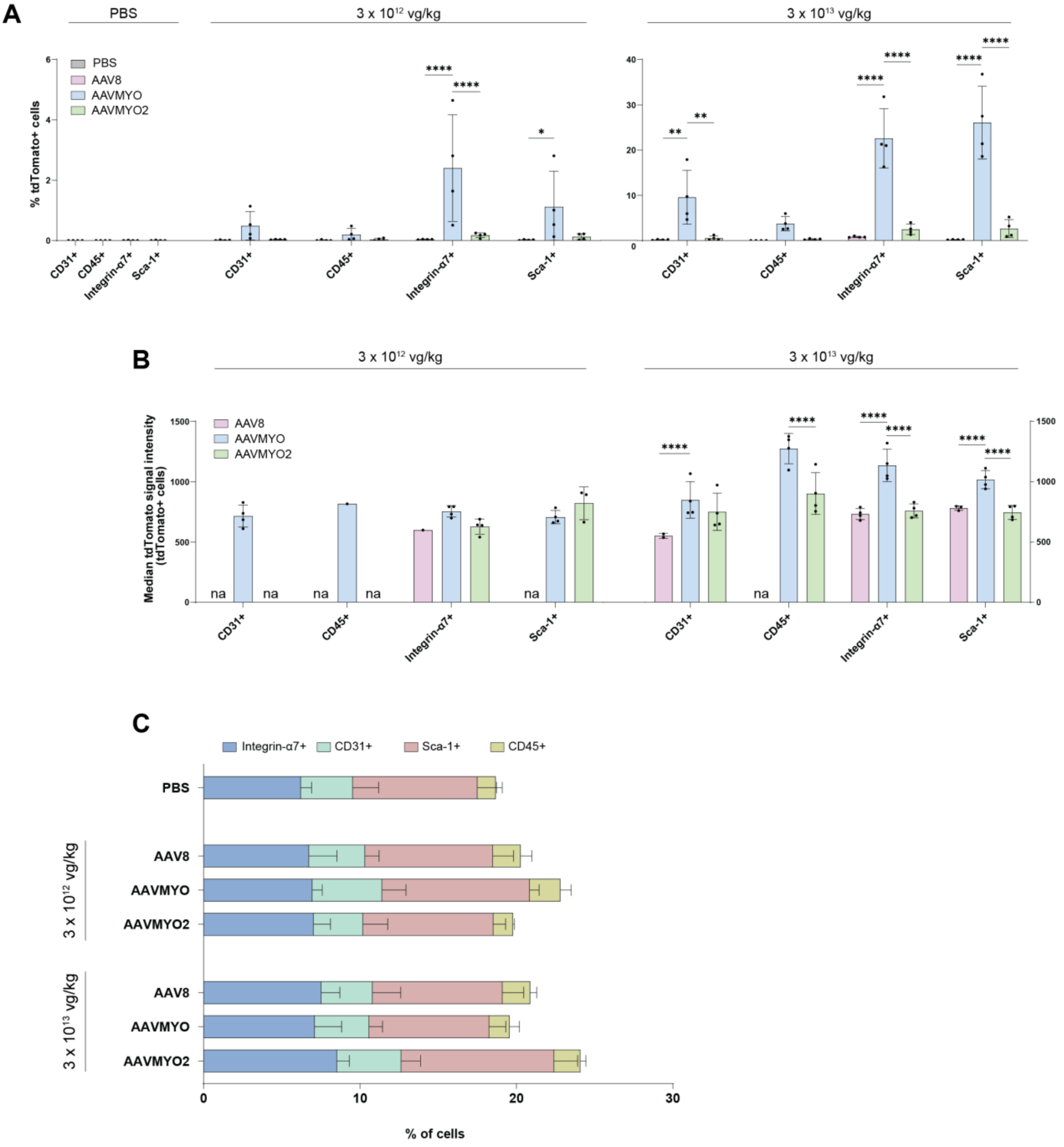
A
PBS
3 x 10^12 vg/kg
3 x 10^13 vg/kg
% tdTomato+ cells
PBS
AAV8
AAVMYO
AAVMYO2
CD31+
CD45+
Integrin-α7+
Sca-1+
B
Median tdTomato signal intensity (tdTomato+ cells)
na
C
Integrin-α7+
CD31+
Sca-1+
CD45+
PBS
AAV8
AAVMYO
AAVMYO2
% of cells

Fig. S3: Transduction rates and effect on muscle composition of IV-injected AAV8, AAVMYO and AAVMYO2. **A** Quantification of the proportion of tdTomato+ cells in CD31+, CD45+, Integrin-α7+ and Sca-1+ cells. **B** Quantification of the median tdTomato signal intensity in CD31+/tdTomato+, CD45+/tdTomato+, Integrin-α7+/tdTomato+ and Sca-1+/tdTomato+ cells. na = not applicable, when < 5 tdTomato+ cells were detected in the cell population, the median tdTomato signal was not measured. **C** Quantification of the proportion of CD31+, CD45+, Integrin-α7+ and Sca-1+ cells detected during flow cytometry. $n = 4$. Data are shown as means ± SD. In **A** and **B**, statistics were evaluated using one-way ANOVAs with Tukey's multiple comparisons test. In **C**, statistics were evaluated using two-way ANOVAs with Tukey's multiple comparisons test. $p < 0.05$ = *, $p < 0.01$ = **, $p < 0.001$ = ***, $p < 0.001$ = ****.

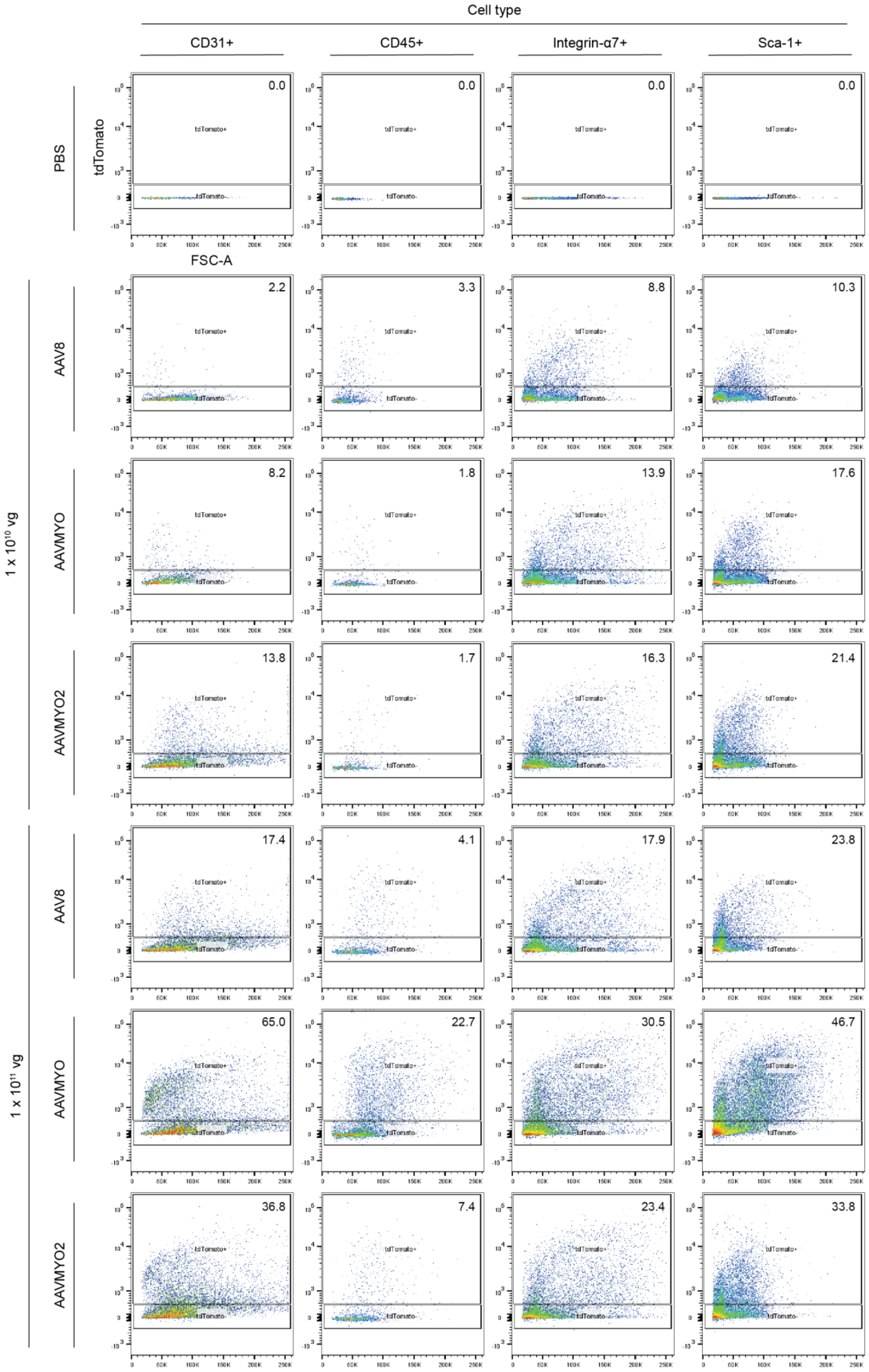

Cell type
CD31+
CD45+
Integrin-α7+
Sca-1+
PBS
tdTomato
FSC-A
tdTomato+
tdTomato-
1 x 10^10 vg
AAV8
AAVMYO
AAVMYO2
1 x 10^11 vg
0.0
0.0
0.0
0.0
2.2
3.3
8.8
10.3
8.2
1.8
13.9
17.6
13.8
1.7
16.3
21.4
17.4
4.1
17.9
23.8
65.0
22.7
30.5
46.7
36.8
7.4
23.4
33.8

Fig. S4: Flow cytometry panel after IM injection of AAV8, AAVMYO and AAVMYO2. Representative panels showing tdTomato signal in CD31+, CD45+, Integrin-α7+ and Sca-1+ cells (the staining strategy to isolate these cells is shown in Fig. S1) of mice injected IM with PBS, AAV8, AAVMYO or AAVMYO2 with 1.0×10^{10} or 1.0×10^{11} vg per TA. The average proportion of tdTomato+ cells is displayed in the top right corner of each panel.

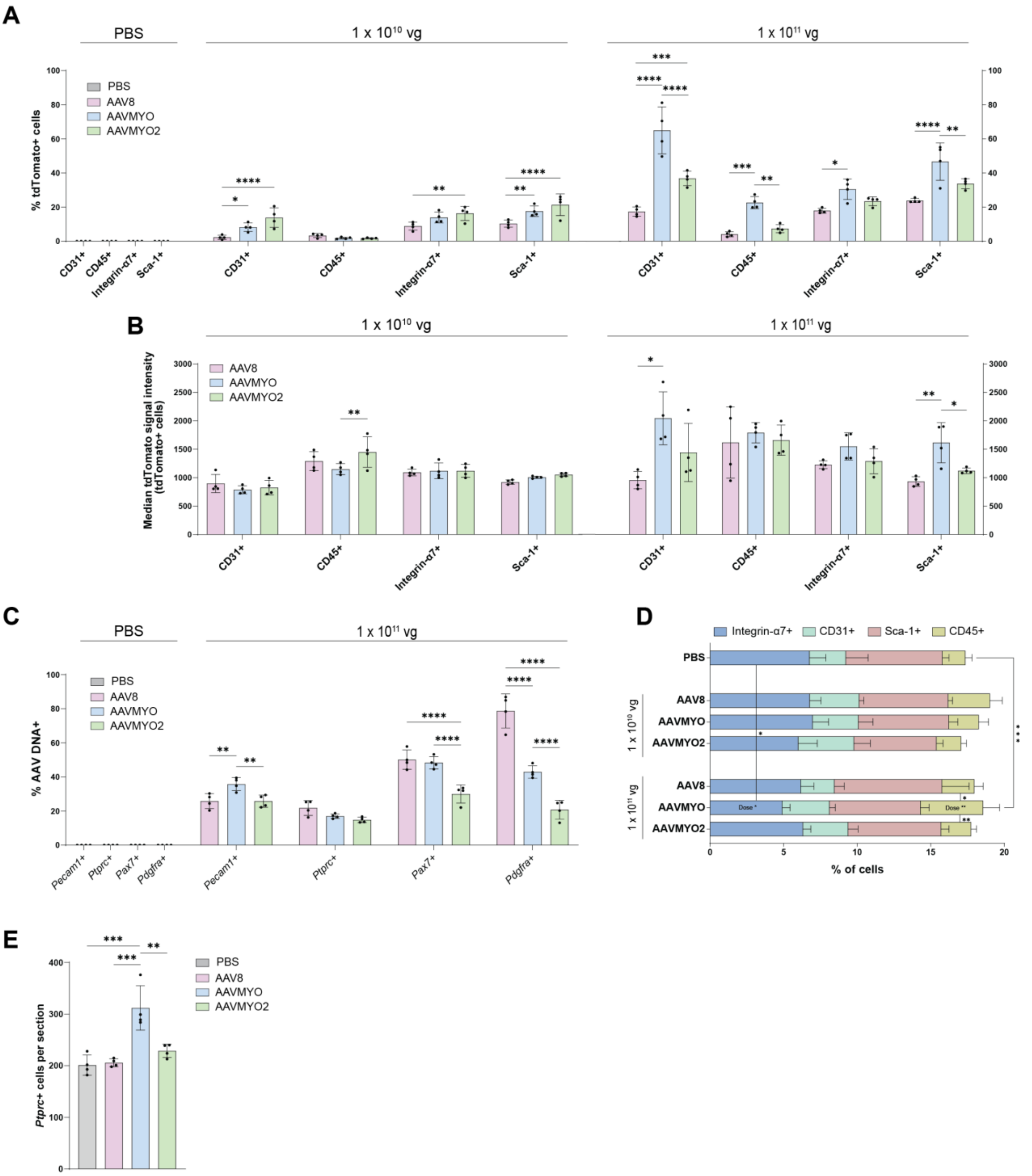
A
PBS
1 x 10^10 vg
1 x 10^11 vg
% tdTomato+ cells
PBS
AAV8
AAVMYO
AAVMYO2
CD31+
CD45+
Integrin-α7+
Sca-1+
B
1 x 10^10 vg
1 x 10^11 vg
Median tdTomato signal intensity (tdTomato+ cells)
AAV8
AAVMYO
AAVMYO2
CD31+
CD45+
Integrin-α7+
Sca-1+
C
PBS
1 x 10^11 vg
% AAV DNA+
PBS
AAV8
AAVMYO
AAVMYO2
Pecam1+
Ptprc+
Pax7+
Pdgfra+
D
Integrin-α7+
CD31+
Sca-1+
CD45+
PBS
1 x 10^10 vg
AAV8
AAVMYO
AAVMYO2
1 x 10^11 vg
AAV8
AAVMYO
AAVMYO2
Dose *
Dose **
% of cells
E
Ptprc+ cells per section
PBS
AAV8
AAVMYO
AAVMYO2

Fig. S5: Transduction rates and effect on muscle composition of IM-injected AAV8, AAVMYO and AAVMYO2. **A** Quantification of the proportion of tdTomato+ cells in CD31+, CD45+, Integrin-α7+ and Sca-1+ cells. **B** Quantification of the median tdTomato signal intensity in CD31+/tdTomato+, CD45+/tdTomato+, Integrin-α7+/tdTomato+ and Sca-1+/tdTomato+ cells. **C** Quantification of the proportion of cell types co-localizing with AAV DNA by smFISH. For each cell type, the proportion of AAV DNA+ cells was calculated by dividing the number of DAPI+ nuclei co-localizing with both AAV DNA and the cell-specific marker by the total number of DAPI+ nuclei co-localizing with the cell-specific marker. **D** Quantification of the proportion of CD31+, CD45+, Integrin-α7+ and Sca-1+ cells detected during flow cytometry. n = 4. **E** Quantification of the number of *Ptprc*-expressing immune cells per cross-section by smFISH. Data are shown as means ± SD. In **A**, **B** and **C**, statistics were evaluated using one-way ANOVAs with Tukey's multiple comparisons test. In **D**, statistics were evaluated using two-way ANOVAs with Tukey's multiple comparisons test and dose effects are shown as Dose* inside of the bar at the higher titer. In **E**, statistics were evaluated using an unpaired student's t-test. $p < 0.05$ = *, $p < 0.01$ = **, $p < 0.001$ = ***, $p < 0.001$ = ****.

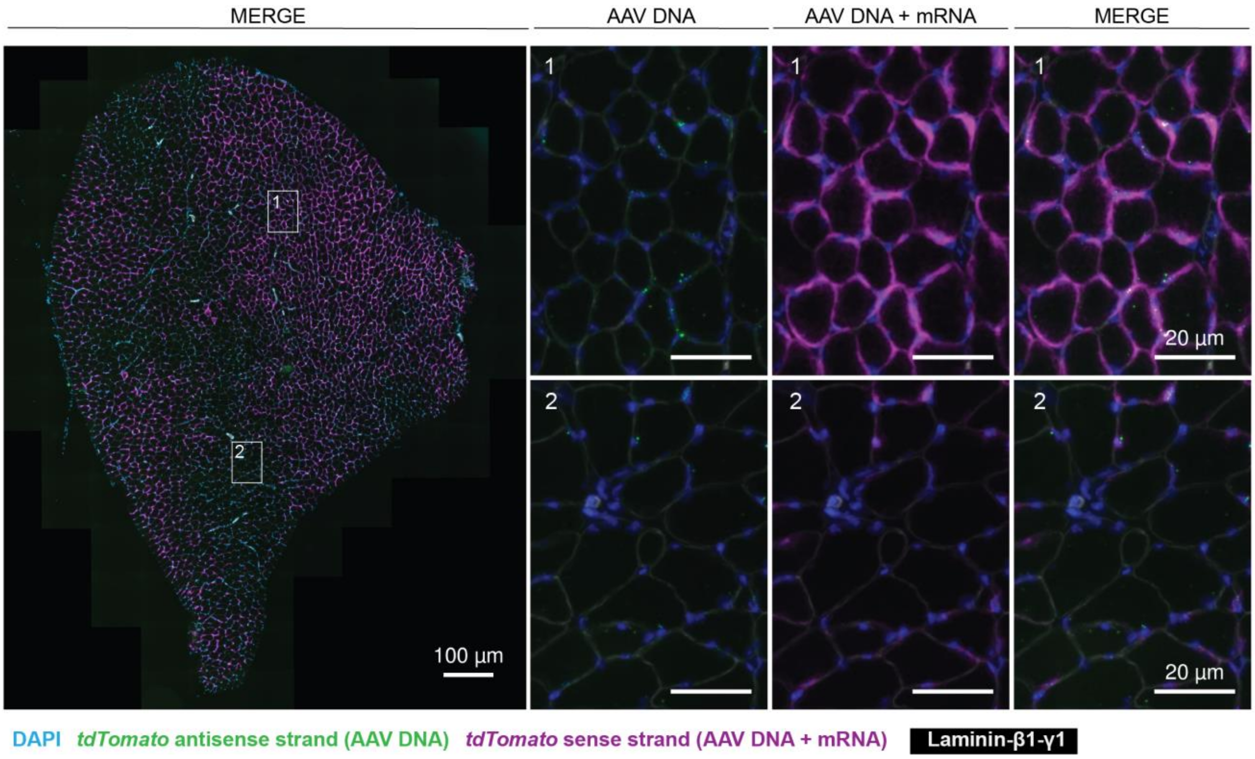

Fig. S6: Probes against *tdTomato* sense and antisense strands detect *tdTomato* DNA and mRNA post-AAV administration. smFISH of full TA section shows the presence of *tdTomato* sense (magenta) and antisense (green) strands in the muscle 3 weeks post-IM administration of 1 x 10^{10} vg per TA of AAVMYO. The probe for the *tdTomato* antisense strand is specific to AAV DNA, while the probe for the sense strand detects AAV DNA and mRNA. Box 1 highlights an area of high AAV transduction, box 2 shows an area of the same muscle with low transduction. Laminin-β1-γ1 is stained in white, DAPI in blue.

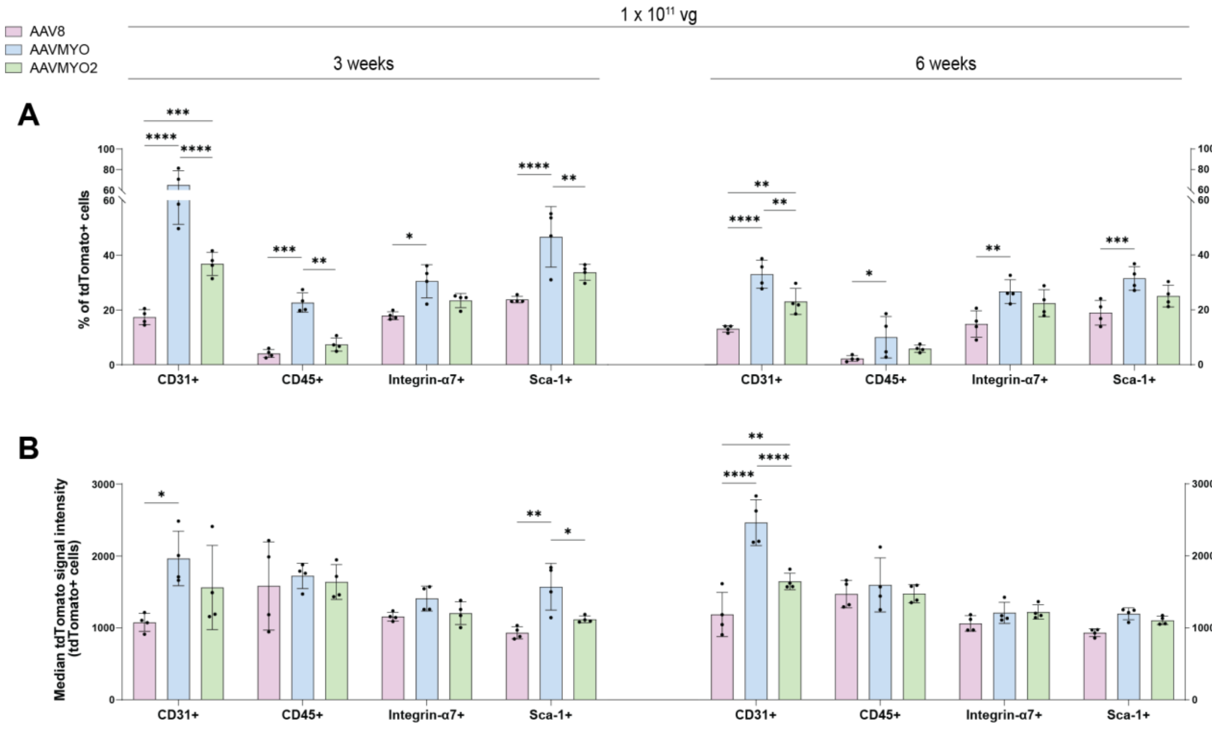

<u>Fig. S7: Quantification of AAV8, AAVMYO and AAVMYO2 transduction levels 3 and 6 weeks post-IM administration</u>. **A** Quantification of the proportion of tdTomato+ cells in CD31+, CD45+, Integrin-α7+ and Sca-1+ cells 3 and 6 weeks post-administration of 1 x 10^{11} vg per TA. **B** Quantification of the median tdTomato signal intensity in CD31+/tdTomato+, CD45+/tdTomato+, Integrin-α7+/tdTomato+ and Sca-1+/tdTomato+ cells 3 and 6-weeks post AAV-administration. Data are shown as means ± SD. Statistics were evaluated for each cell type using one-way ANOVAs with Tukey's multiple comparisons test. $p < 0.05$ = *, $p < 0.01$ = **, $p < 0.001$ = ***, $p < 0.001$ = ****.

Table S1: Flow cytometry quantifications of the proportion of cell types and tdTomato+ cells after IV injection.

	% of single cells				% tdTomato+			
Treatment	CD31	CD45	Integrin-a7	Sca-1	CD31	CD45	Integrin-a7	Sca-1
PBS	4.58	0.83	5.2	6.86	0	0	0	0
AAV8 3E+12 vg/kg	4.44	0.92	4.88	6.85	0	0	0.04	0.038
AAVMYO 3E+12 vg/kg	2.3	1.15	6.26	8.57	1.14	0.21	4.65	1.01
AAVMYO2 3E+12 vg/kg	1.93	1.23	6.69	7.58	0.038	0	0.18	0.02
PBS	2.75	1.65	6.67	9.24	0	0	0	0
AAV8 3E+12 vg/kg	4.24	1.47	8.69	8.06	0.013	0.038	0.045	0.014
AAVMYO 3E+12 vg/kg	4.58	1.64	7.56	9.79	0.21	0.062	1.64	0.53
AAVMYO2 3E+12 vg/kg	2.47	1.14	7.33	7.81	0.024	0.053	0.21	0.21
PBS	4.72	0.85	6.38	7	0	0	0	0
AAV8 3E+12 vg/kg	2.61	2.26	7.78	9.97	0.027	0	0.027	0.028
AAVMYO 3E+12 vg/kg	5.11	2.46	6.58	9.56	0.074	0.038	0.51	0.13
AAVMYO2 3E+12 vg/kg	2.8	1.34	8.35	9.27	0.042	0.088	0.063	0.057
PBS	1.24	1.36	6.63	8.77	0	0	0.013	0.019
AAV8 3E+12 vg/kg	3.08	2.43	5.59	7.84	0	0	0.037	5.32E-03
AAVMYO 3E+12 vg/kg	5.84	2.59	7.42	9.84	0.53	0.48	2.81	2.81
AAVMYO2 3E+12 vg/kg	5.45	1.3	5.79	8.65	0.032	0	0.25	0.22
AAV8 3E+13 vg/kg	2.73	1.22	6.27	7.78	0.22	0	1.02	0.21
AAVMYO 3E+13 vg/kg	2.67	1.01	6.25	7.34	5.95	2.53	21.3	21.4
AAVMYO2 3E+13 vg/kg	5.11	2.12	8.27	11.6	0.23	0.2	2.58	1.17
AAV8 3E+13 vg/kg	2.91	1.73	6.81	7.33	0.15	0	0.7	0.24
AAVMYO 3E+13 vg/kg	3.53	0.82	6.55	7.61	9.73	5.96	31.8	36.8
AAVMYO2 3E+13 vg/kg	4.13	1.76	7.72	8.49	1.23	0.46	3.99	5.2
AAV8 3E+13 vg/kg	5.84	2.16	8.25	7.8	0.057	0	0.72	0.22
AAVMYO 3E+13 vg/kg	4.65	1.16	5.96	6.62	4.64	2.64	16.3	18.6
AAVMYO2 3E+13 vg/kg	4.81	1.55	8.53	8.58	0.18	0.15	1.32	0.93
AAV8 3E+13 vg/kg	1.62	2	8.79	10.3	0.27	0	0.78	0.14
AAVMYO 3E+13 vg/kg	3.06	2.22	9.66	9.14	17.9	3.76	21	27.5
AAVMYO2 3E+13 vg/kg	2.38	1.29	9.61	10.4	0.52	0.34	1.94	3.2

Table S2: Flow cytometry quantifications of the median tdTomato signal and number of tdTomato+ cells after IV injection.

	Median tdTomato signal				Number of tdTomato+ cells			
Treatment	CD31	CD45	Integrin-a7	Sca-1	CD31	CD45	Integrin-a7	Sca-1
PBS	n/a	n/a	n/a	n/a	0	0	0	0
AAV8 3E+12 vg/kg	n/a	n/a	546	1373	0	0	3	4
AAVMYO 3E+12 vg/kg	738	500	793	674	43	4	475	141
AAVMYO2 3E+12 vg/kg	650	n/a	639	658	1	0	16	2
PBS	n/a	n/a	n/a	n/a	0	0	0	0
AAV8 3E+12 vg/kg	525	540	599	537	1	1	7	2
AAVMYO 3E+12 vg/kg	828	512	732	661	19	2	246	102
AAVMYO2 3E+12 vg/kg	16652	512	642	893	1	1	26	27
PBS	n/a	n/a	n/a	n/a	0	0	0	0
AAV8 3E+12 vg/kg	8794	n/a	571	801	1	0	3	4
AAVMYO 3E+12 vg/kg	611	527	699	708	8	2	72	26
AAVMYO2 3E+12 vg/kg	677	494	541	908	2	2	9	9
PBS	n/a	n/a	522	732	0	0	2	4
AAV8 3E+12 vg/kg	n/a	n/a	546	544	0	0	5	1
AAVMYO 3E+12 vg/kg	685	817	794	784	45	18	303	402
AAVMYO2 3E+12 vg/kg	630	n/a	690	665	3	0	25	32
AAV8 3E+13 vg/kg	567	n/a	789	796	8	0	86	22
AAVMYO 3E+13 vg/kg	1067	1278	1150	941	254	41	2133	2510
AAVMYO2 3E+13 vg/kg	650	755	781	708	22	8	397	252
AAV8 3E+13 vg/kg	507	n/a	679	525	1	0	11	4
AAVMYO 3E+13 vg/kg	840	1097	1320	1112	744	106	4517	6064
AAVMYO2 3E+13 vg/kg	740	905	830	793	108	17	652	935
AAV8 3E+13 vg/kg	929	n/a	720	760	5	0	89	26
AAVMYO 3E+13 vg/kg	747	1348	1026	976	316	45	1421	1808
AAVMYO2 3E+13 vg/kg	641	803	703	685	23	6	299	212
AAV8 3E+13 vg/kg	537	n/a	743	788	8	0	125	26
AAVMYO 3E+13 vg/kg	743	1376	1049	1038	790	120	2917	3611
AAVMYO2 3E+13 vg/kg	972	1145	720	798	17	6	257	460

Table S3: Flow cytometry quantifications of the proportion of cell types and tdTomato+ cells 3 weeks after IM injection.

	% of single cells				% tdTomato+			
Treatment	CD31	CD45	Integrin-a7	Sca-1	CD31	CD45	Integrin-a7	Sca-1
PBS	0.85	2.12	8.17	6.91	0	0	0	0
AAV8 1E+10 vg	3.24	2.47	7.6	5.93	0.78	4.28	9.88	11.1
AAVMYO 1E+10 vg	3.26	2.73	8.56	6.26	5.15	1.29	18.2	22
AAVMYO2 1E+10 vg	2.96	2.09	7.93	5.79	20.5	2.44	17.1	26
PBS	1.75	1.04	5.48	6.1	0	0	0	0
AAV8 1E+10 vg	3.35	2.51	7.11	6.18	3.9	3.84	11.2	12.7
AAVMYO 1E+10 vg	3.93	1.15	6.22	5.44	8.09	2.5	14.4	17.4
AAVMYO2 1E+10 vg	3.23	1.48	5.4	5.4	7.15	2.93	10.3	12
PBS	2.96	1.39	6.94	6.98	0	0	0	0
AAV8 1E+10 vg	3.05	4.09	6.59	6.39	2.12	3.36	5.69	7.92
AAVMYO 1E+10 vg	1.67	2	6.72	6.01	11.3	2.08	11.6	15.1
AAVMYO2 1E+10 vg	3.51	1.84	5.28	6.13	11.7	1.87	20	22.9
PBS	4.33	1.68	6.47	6.26	0	0	0	7.31E-03
AAV8 1E+10 vg	3.8	2.29	5.83	5.7	2.17	1.56	8.4	9.52
AAVMYO 1E+10 vg	3.55	2.25	6.43	6.91	8.19	1.39	11.3	15.8
AAVMYO2 1E+10 vg	5.41	1.31	5.42	5.14	15.8	2.97	17.8	24.5
AAV8 1E+11 vg	1.81	1.99	7.44	9.69	14.5	2.5	17.1	23.1
AAVMYO 1E+11 vg	3.24	4.95	5.28	6.76	70.5	27.4	36.1	47
AAVMYO2 1E+11 vg	2.49	1.75	6.54	6.31	36.3	6.75	24.5	36.5
AAV8 1E+11 vg	3.26	3.09	5.37	6.78	15.8	5.98	19.9	25.6
AAVMYO 1E+11 vg	3.39	4.32	5.44	6.6	81.2	20.1	33.3	55.1
AAVMYO2 1E+11 vg	3.65	2.22	5.36	6.03	41.6	4.88	24.5	35
AAV8 1E+11 vg	1.88	1.98	5.83	5.27	18.6	3.4	18	23.3
AAVMYO 1E+11 vg	2.65	2.63	4.56	5.89	49.7	19.8	22.1	31
AAVMYO2 1E+11 vg	2.49	2.47	5.53	7.07	31.4	7.23	25.1	33.7
AAV8 1E+11 vg	2.19	1.74	6.05	7.53	20.5	4.53	16.6	23.2
AAVMYO 1E+11 vg	3.55	5.08	4.38	5.52	58.6	23.3	30.4	53.6
AAVMYO2 1E+11 vg	3.65	1.74	5.04	5.87	38	10.6	19.5	29.7

Table S4: Flow cytometry quantifications of the median tdTomato signal and number of tdTomato+ cells 3 weeks after IM injection.

	Median tdTomato signal				Number of tdTomato+ cells			
Treatment	CD31	CD45	Integrin-a7	Sca-1	CD31	CD45	Integrin-a7	Sca-1
PBS	n/a	n/a	n/a	n/a	0	0	0	0
AAV8 1E+10 vg	1136	1368	1177	901	41	171	1216	1068
AAVMYO 1E+10 vg	807	1051	1320	1032	204	43	1900	1680
AAVMYO2 1E+10 vg	862	1123	1246	1018	1231	103	2742	3048
PBS	n/a	n/a	n/a	n/a	0	0	0	0
AAV8 1E+10 vg	805	1489	1069	946	246	182	1505	1481
AAVMYO 1E+10 vg	737	1270	1108	1010	730	66	2062	2179
AAVMYO2 1E+10 vg	740	1484	1065	1032	351	66	841	979
PBS	n/a	n/a	n/a	n/a	0	0	0	0
AAV8 1E+10 vg	846	1179	1084	879	132	281	765	1033
AAVMYO 1E+10 vg	879	1177	1046	1004	456	101	1898	2206
AAVMYO2 1E+10 vg	994	1421	1177	1078	883	74	2264	3014
PBS	n/a	n/a	n/a	738	0	0	0	1
AAV8 1E+10 vg	807	1127	1036	964	148	64	881	976
AAVMYO 1E+10 vg	727	1101	1006	984	476	51	1187	1786
AAVMYO2 1E+10 vg	713	1777	996	1080	2005	91	2257	2945
AAV8 1E+11 vg	1072	945	1235	846	515	97	2490	4389
AAVMYO 1E+11 vg	2006	1758	1544	1501	5933	3521	4948	8255
AAVMYO2 1E+11 vg	2410	1432	1381	1093	1572	206	2792	4014
AAV8 1E+11 vg	912	1987	1143	880	835	299	1728	2803
AAVMYO 1E+11 vg	2485	1877	1570	1801	7714	2435	5073	10196
AAVMYO2 1E+11 vg	1190	1459	1172	1186	2957	211	2562	4109
AAV8 1E+11 vg	1104	1183	1156	1032	1055	204	3185	3717
AAVMYO 1E+11 vg	1709	1470	1258	1141	3719	1471	2849	5164
AAVMYO2 1E+11 vg	1489	1946	1256	1112	1879	429	3333	5730
AAV8 1E+11 vg	1213	2214	1089	968	1071	189	2402	4177
AAVMYO 1E+11 vg	1661	1788	1256	1839	8286	4714	5305	11795
AAVMYO2 1E+11 vg	1156	1715	1004	1082	3294	437	2330	4144

Table S5: Flow cytometry quantifications of the proportion of cell types and tdTomato+ cells 6 weeks after IM injection.

	% of single cells				% tdTomato+			
Treatment	CD31	CD45	Integrin-a7	Sca-1	CD31	CD45	Integrin-a7	Sca-1
AAV8 1E+11 vg	1.34	2.8	7.18	9.73	14	1.1	20.6	24.1
AAVMYO 1E+11 vg	1.61	3.1	7.05	7.92	38.7	4.69	32.6	36.8
AAVMYO2 1E+11 vg	2.58	2.36	7.15	8.07	29.7	7.5	28.8	30.3
AAV8 1E+11 vg	1.54	3.02	8.37	8.05	14	3.7	15.9	21.1
AAVMYO 1E+11 vg	1.05	1.48	6.15	6.37	27.7	2.77	26	27.1
AAVMYO2 1E+11 vg	1.08	1.21	6.33	6.15	22.3	5.5	23.7	25.8
AAV8 1E+11 vg	1.05	1.6	4.8	4.57	11.5	2.05	9	13.9
AAVMYO 1E+11 vg	2.1	4.77	6.76	6.9	29.8	18.6	22.2	28.9
AAVMYO2 1E+11 vg	1.88	3.2	8.47	11.2	18.5	4.35	19.3	21.2
AAV8 1E+11 vg	1.83	2.97	5.76	6.28	12.9	1.99	13.9	16.8
AAVMYO 1E+11 vg	1.34	1.93	5.75	7.78	35.7	14	25.9	33.1
AAVMYO2 1E+11 vg	2.85	1.62	8.7	8.07	21.8	5.94	17.9	22.9

Table S6: Flow cytometry quantifications of the median tdTomato signal and number of tdTomato+ cells 6 weeks after IM injection

	Median tdTomato signal				Number of tdTomato+ cells			
Treatment	CD31	CD45	Integrin-a7	Sca-1	CD31	CD45	Integrin-a7	Sca-1
AAV8 1E+11 vg	1612	1290	1183	970	437	72	3444	5457
AAVMYO 1E+11 vg	2832	2123	1424	1213	1208	282	4458	5665
AAVMYO2 1E+11 vg	1815	1576	1363	1172	945	218	2537	3014
AAV8 1E+11 vg	1183	1320	1117	972	394	203	2419	3095
AAVMYO 1E+11 vg	2189	1251	1132	1074	653	92	3595	3872
AAVMYO2 1E+11 vg	1570	1591	1172	1059	534	147	3318	3500
AAV8 1E+11 vg	903	1606	968	858	274	74	974	1433
AAVMYO 1E+11 vg	2624	1440	1108	1244	1004	1421	2412	3203
AAVMYO2 1E+11 vg	1621	1384	1202	1132	1049	419	4928	7150
AAV8 1E+11 vg	1044	1661	970	927	392	98	1327	1746
AAVMYO 1E+11 vg	2202	1570	1168	1251	1226	690	3813	6596
AAVMYO2 1E+11 vg	1573	1350	1141	1051	1107	171	2765	3288

Table S7: List of antibodies used.

Antibody	Company	Reference	Dilution
Rat anti-CD31, Alexa Fluor 700 conjugated	BioLegend (San Diego, California)	102444	1/200
Rat anti-CD45, Alexa Fluor 488 conjugated	BioLegend (San Diego, California)	103122	1/400
Rat anti-Integrin-α7, Alexa Fluor 647 conjugated	Ablab (Sao Paulo, Brazil)	67-0010-05	1/1000
Rat anti-Sca-1, Bright Violet 421 conjugated	BioLegend (San Diego, California)	108128	1/400
Anti-Laminin-β1-γ1 produced in rabbit	Sigma-Aldrich (St. Louis, Missouri)	L9393	1/100
Goat anti-Rabbit IgG (H+L) Cross-Absorbed Secondary Antibody, Alexa Fluor 750	Thermo Fisher Scientific (Waltham, Massachusetts)	A-21039	1/100

Table S8: List of RNAscope probes used.

Probe	Channel	Catalog Number
tdTomato antisense (to detect sense sequence)	C1	317041
Probe diluent	C1	300041
tdTomato sense (to detect antisense sequence)	C2	539641-C2
Pdgfra	C3	480661-C3
Pecam1	C3	316721-C3
Ptprc	C3	318651-C3
Pax7	C3	314181-C3
